# Occurrence of metabolic syndrome in young adults and inequalities according to sex, skin color, and socioeconomic status: evidence from two birth cohorts in Southern Brazil

**DOI:** 10.1590/0102-311XEN178025

**Published:** 2026-04-27

**Authors:** Ana Paula Oliveira Rosses, Gabriela Ávila Marques, Bruna Gonçalves-Silva, Ana M. B. Menezes, Helen Gonçalves, Fernando Hartwig, Bernardo L. Horta, Fernando C. Wehrmeister

**Affiliations:** 1 Programa de Pós-graduação em Epidemiologia, Universidade Federal de Pelotas, Pelotas, Brasil.

**Keywords:** Metabolic Syndrome, Cardiometabolic Risk Factors, Health Status Disparities, Cohort Studies, Síndrome Metabólico, Factores de Riesgo Cardiometabólico, Desigualdades en la Salud, Estudios de Cohortes

## Abstract

The aim of the present study was to identify the prevalence of metabolic syndrome at 22 and 30 years of age, determine inequalities by sex, skin color, and socioeconomic status, and investigate the intersections of these variables. A cross-sectional analysis was conducted using data from the 1993 (22 years of age) and 1982 (22 and 30 years of age) Pelotas (Brazil) birth cohort. Metabolic syndrome was defined based on the criteria of the *National Cholesterol Education Program Adult Treatment Panel III* (NCEP-ATP III) and the International Diabetes Federation (IDF). Inequalities in terms of sex, skin color, and socioeconomic status were described in Equiplots and analyzed by absolute measures (prevalence ratio and slope index of inequality - SII]) and relative measures (difference in prevalence and concentration index - CIX]). At 22 years of age, the prevalence of metabolic syndrome was 18.2% (IDF) and 13.5% (NCEP-ATP III) in the 1993 cohort, and 11.3% (IDF) and 8.6% (NCEP-ATP III) in the 1982 cohort. At 30 years of age (1982 cohort), the prevalence was 15.5% (IDF) and 10.7% (NCEP-ATP III). The greatest inequality in terms of socioeconomic status occurred in the 1982 cohort at 22 years of age based on the NCEP-ATP III criteria among women (SII: -8.7p.p. [95%CI: -13.2; -4.2]; CIX: -20.1% [95%CI: -29.7; -10.5]) and men (SII: 6.5p.p. [95%CI: 0.1; 12.9]; CIX: 7.9% [95%CI: 0.6; 15.3]). The most affected subgroups were White women with a lower socioeconomic status and Black/Mixed-race men (1993 cohort) and White men (1982 cohort) with a higher socioesconomic status. Our results demonstrate that metabolic syndrome is not evenly distributed in this population and that its occurrence may be influenced by intersections between socioeconomic status, sex, and skin color.

## Abstract (es)

El objetivo de este estudio fue identificar la prevalencia del síndrome metabólico a los 22 y 30 años, y las desigualdades por sexo, color de piel y nivel socioeconómico, y sus intersecciones. Se trata de un análisis transversal que utiliza datos de las cohorte de nacimientos de Pelotas, Rio Grande do Sul, Brasil, de 1993 (22 años) y 1982 (22 y 30 años). El síndrome metabólico se definió según los criterios de la *National Cholesterol Education Program Adult Treatment Panel III* (NCEP-ATP III) y de la Federación Internacional de Diabetes (IDF). Las desigualdades entre sexo, color de piel y nivel socioeconómico se describieron en Equiplots y se analizaron utilizando medidas absolutas (diferencia de prevalencia y índice de pendiente de desigualdad – SII) y medidas relativas (razón de prevalencia e índice de concentración – CIX]). A los 22 años, la prevalencia del síndrome metabólico fue del 18,2% (IDF) y del 13,5% (NCEP-ATP III) (cohorte de 1993), y del 11,3% (IDF) y del 8,6% (NCEP-ATP III) (cohorte de 1982). A los 30 años (cohorte de 1982), fue del 15,5% (IDF) y del 10,7% (NCEP-ATP III). La mayor desigualdad por nivel socioeconómico se dio en la cohorte de 1982 a los 22 años, según el NCEP-ATP III: entre mujeres (SII: -8,7p.p.; [IC95%: -13,2; -4,2]; CIX: -20,1%; [IC95%: -29,7; -10,5]) y hombres (SII: 6,5p.p.; [IC95%: 0,1; 12,9]; CIX: 7,9%; [IC95%: 0,6; 15,3]). Los subgrupos más afectados fueron: mujeres blancas con menor nivel socioeconómico, hombres negros/pardos (cohorte de 1993) y hombres blancos (cohorte de 1982) con mayor nivel socioeconómico. Nuestros resultados refuerzan que el síndrome metabólico no está distribuido de forma homogénea en esta población y que su aparición puede estar influenciada por intersecciones entre el nivel socioeconómico, el sexo y el color de la piel.

## Introduction

Cardiometabolic diseases have been the leading cause of global mortality since the 1940s and currently account for approximately one-third of deaths throughout the world ^1^ ^,^ ^2^ ^,^ ^3^, corresponding to about 20 million deaths per year, 80% of which occur in low- and middle-income countries ^4^. Cardiometabolic diseases have progressively contributed to the increase in health-related costs over the years both directly, due to hospitalizations, follow-ups, procedures, and treatments, and indirectly, due to the loss of productivity ^5^ ^,^ ^6^ ^,^ ^7^ ^,^ ^8^.

Metabolic syndrome is a set of metabolic alterations that increase the risk of cardiometabolic diseases. The most widely used criteria for the diagnosis of metabolic syndrome are those of the *National Cholesterol Education Program Adult Treatment Panel III* (NCEP-ATP III) and International Diabetes Federation (IDF) ^9^. The diagnosis of metabolic syndrome is established by both sets of criteria when at least three of the following five factors are present: central obesity (waist circumference ≥ 102/88cm for men/women according to the NCEP-ATP III or 90/80cm according to the IDF), high blood pressure (≥ 135/85mmHg), dyslipidemia (triglycerides ≥ 150mg/dL or HDL-cholesterol < 40mg/dL in men and < 50mg/dL in women), and high fasting glucose (≥ 100mg/dL) or diabetes ^9^.

Studies have shown that the presence of metabolic syndrome practically doubles the risk of cardiovascular disease ^10^ ^,^ ^11^ ^,^ ^12^ ^,^ ^13^ and is also recognized as a robust predictor for the development of type 2 diabetes, increasing the risk up to fivefold in adults ^14^ ^,^ ^15^ ^,^ ^16^. Although cardiometabolic diseases generally manifest clinically in the older population, metabolic syndrome and its components have been established increasingly earlier in adulthood, with evidence pointing to their origin in childhood and adolescence ^17^.

Metabolic syndrome and cardiometabolic diseases have a multifactorial etiology and are influenced by biological, behavioral, and socioeconomic factors ^18^. Social inequality influences both the emergence and progression of these conditions. Individuals with greater vulnerability, such as those with a low income, lower education, and those belonging to racial or ethnic minorities, are at greater risk of being affected ^19^ ^,^ ^20^ ^,^ ^21^. The relationship between socioeconomic status and the risk of metabolic syndrome and cardiometabolic diseases differs on the global scale. The pattern is typically linear and well-established in high-income countries, where low socioeconomic status is consistently associated with a greater risk of cardiometabolic diseases ^1^ ^,^ ^18^ ^,^ ^22^. In low- and middle-income countries, however, the pattern is more complex due to the epidemiological transition, by which the greater risk of cardiometabolic diseases initially affects the strata with the best socioeconomic status and tends to migrate to the social base with socioeconomic advance ^23^ ^,^ ^24^ ^,^ ^25^.

As a middle-income country historically marked by profound inequities, Brazil is part of this context of epidemiological transition ^24^ ^,^ ^26^ ^,^ ^27^. Thus, the direct application of findings from high-income countries to Brazil is limited, requiring a detailed understanding of how structural inequalities related to skin color and sex interact with socioeconomic status to determine the risk of metabolic syndrome ^28^. Therefore, the aim of the present study was to estimate the prevalence of metabolic syndrome in individuals 22 and 30 years of age in follow-up assessment of the 1982 and 1993 Pelotas (Brazil) birth cohorts and analyze inequalities of its occurrence in terms of sex, skin color, and socioeconomic status.

## Methods

### Design and setting

Cross-sectional analyses were conducted using data from the 1982 and 1993 Pelotas (Brazil) birth cohorts. Pelotas is a medium-sized municipality in the Southern most portion of Brazil with a population of 325,685 in 2022, 23.9% of whom are self-declared Black/Mixed-race ^29^ ^,^ ^30^. 

All children born in hospitals between January 1 and December 31 in 1982 and 1993 whose families lived in urban areas of the municipality of Pelotas were part of the cohorts. The initial sample consisted of 5,914 individuals in 1982 and 5,249 in 1993 ^31^ ^,^ ^32^ ^,^ ^33^ ^,^ ^34^ ^,^ ^35^. 

The analyses were conducted based on three assessments: at 22 and 30 years of age in the 1982 cohort and at 22 years of age in the 1993 cohort. This strategy enabled investigating changes in the same group of individuals as well as comparing different generations at similar ages. 

The participants had been prospectively followed up at different stages of life, with follow-up rates of 77.4% at 22 years of age and 68.1% at 30 years of age in the 1982 cohort and 76.3% at 22 years of age in the 1993 cohort. The collection of information from all follow-ups was carried out by teams trained in a standardized manner for the application of the questionnaires, the measurement of anthropometric variables, and other tests ^31^ ^,^ ^32^ ^,^ ^33^ ^,^ ^34^ ^,^ ^35^. Further details on the methodological aspects of the cohorts have been published previously ^31^ ^,^ ^32^ ^,^ ^33^ ^,^ ^34^ ^,^ ^35^.

### Outcomes

The presence of metabolic syndrome was assessed at 22 and 30 years of age in the 1982 cohort and 22 years of age in the 1993 cohort according to the NCEP-ATP III and IDF criteria. Both consider five components - blood glucose, HDL cholesterol, triglycerides, blood pressure, and waist circumference - and the diagnosis of metabolic syndrome is established when at least three of these five parameters are altered. The main difference between the sets of criteria regards the cutoff point adopted for waist circumference, which is more restrictive in the IDF criteria. The forms of collection, cutoff points, and operationalization of the variables are detailed in Box 1.

**Box 1:** Outcome variables and criteria for defining metabolic syndrome according to *National Cholesterol Education Program Adult Treatment Panel III* (NCEP-ATP III) and International Diabetes Federation (IDF) in 1982 (22 and 30 years) and 1993 (22 years) Pelotas (Brazil) birth cohorts.

VARIABLES AND CRITERIA	1982 COHORT		1993 COHORT
	22 YEARS	30 YEARS	22 YEARS
Central obesity	Standardized measurement with non-elastic tape measure. Average of two measurements at midpoint between last rib and iliac bone. Altered when waist circumference ≥ 102/88cm (NCEP-ATP III) and 90/80cm (IDF) for men/women		
Blood pressure	Measured with digital device (Omron HEM-629). Average of two resting measurements of at least five minutes on the right wrist. Altered when ≥ 130/85mmHg or self-reported use of antihypertensive medication	Measured with digital device (Omron HEM-705CPINT). Two standardized measurements at beginning and end of anthropometric assessment on left arm, with participant seated at rest and cuff appropriate to arm circumference. Altered if ≥ 130/85mmHg or use of antihypertensive medication; 1993 cohort also by self-reported medical diagnosis of hypertension	
Blood glucose	Random collection. Assessed by capillary blood glucose using portable glucometer (Accu-Chek Advantage - Roche). Altered if ≥ 100mg/dL and/or self-reported use of diabetes medication	Random collection from vein. Assessed by colorimetric enzymatic method using Selectra 2 (Merck) and BS-380 (Shenzhen Mindray Bio-Medical Electronics Co.) analyzers. Altered if ≥ 100mg/dL or self-reported use of medication for diabetes; 1993 cohort also by self-reported medical diagnosis of diabetes	
Triglycerides	Random collection from vein. Assessed by colorimetric enzymatic method with Selectra 2 (Merck) and BS-380 (Shenzhen Mindray Bio-Medical Electronics Co.) analyzers. Altered if ≥ 150mg/dL or self-reported use of dyslipidemia medication; 1993 cohort also by self-reported medical diagnosis of dyslipidemia		
HDL cholesterol	Random collection from vein. Assessed by ultrasensitive direct method at 22 years of age in 1982 and colorimetric enzymatic method at other follow-ups. Selectra 2 (Merck) and BS-380 (Shenzhen Mindray Bio-Medical Electronics Co.) analyzers used. Altered if ≤ 40mg/dL in men or ≤ 50mg/dL in women or self-reported use of dyslipidemia medication; 1993 cohort also by self-reported medical diagnosis of dyslipidemia		
Metabolic syndrome NCEP-ATP III	Presence of 3 or more altered components: triglycerides ≥ 150mg/dL, HDL < 40mg/dL (men) or < 50mg/dL (women), body pressure ≥ 130/85mmHg, blood glucose ≥ 100mg/dL, and waist circumference ≥ 102cm (men) or ≥ 88cm (women)		
Metabolic syndrome IDF	Presence of 3 or more altered components above, differing only in waist circumference ≥ 90cm (men) or ≥ 80cm (women)		

### Exposures

Information on the sex (male/female) of the participants was collected in the perinatal studies of both cohorts. Skin color was self-declared by the participants using the categories of the Brazilian Institute of Geography and Statistics (IBGE, acronym in Portuguese) at 22 years of age in the 1982 cohort and at 15 years of age in the 1993 cohort.

Socioeconomic status was measured using the classification of the Brazilian Association of Research Companies (ABEP, acronym in Portuguese) ^36^ and grouped into three categories (AB, C, and DE). Schooling in complete years of formal education was categorized as 0-4, 5-8, 9-11, and ≥ 12 years of study. Both measures were determined at the same time as the metabolic syndrome assessment in each cohort: at 22 and 30 years of age in the 1982 cohort and 22 years of age in the 1993 cohort.

### Data analysis 

Pregnant women and participants with missing data as well as individuals of yellow skin color or indigenous individuals were excluded from the analysis due to their small numbers. 

To investigate possible interactions among stratifiers (sex, skin color, socioeconomic status, and schooling), an analysis of the interaction terms was performed in logistic regression models in each cohort, with the occurrence of metabolic syndrome defined by the IDF and NCEP-ATP III criteria as a dichotomous outcome. No evidence of interaction (p < 0.20) was found between socioeconomic status and skin color in any of the follow-ups analyzed. In contrast, interactions were found between sex and the other three stratifiers at all follow-ups. The interaction with the highest p-value was between sex and skin color (p-value = 0.17) using the NCEP-ATP III criteria at 22 years of age in the 1993 cohort, while the other interactions had a p-value < 0.05.

All analyses were performed separately for the NCEP-ATP III and IDF criteria. Measures of absolute and relative inequalities were analyzed. For inequality between the sexes (dichotomous variable), differences in the prevalence of metabolic syndrome and the prevalence ratio was calculated according to the two sets of criteria (NCEP-ATP III and IDF). Proportions were compared using Pearson’s chi-square test, with a p-value < 0.05 considered indicative of a significant difference. 

Equiplots (https://equidade.org/en/equiplot) were created to visualize and identify patterns of inequality. In this type of graph, each point represents the prevalence of the outcome of interest in population subgroups defined by sex and skin color, and the distance between points expresses the magnitude of inequality between the categories analyzed ^37^. 

The slope index of inequality (SII) was used for socioeconomic status (ordinal variable). This measure determines the absolute difference in the prevalence of metabolic syndorme throughout the distribution ^38^. The concentration index (CIX) was used for relative inequality, which quantifies the concentration of a health outcome at different points in the income distribution or other socioeconomic measure ^38^. 

All analyses were performed using the Stata software, version 18.0 (https://www.stata.com), and Excel, version 2505 build 16.0.18827.20102 (https://products.office.com/).

### Ethical aspects

The follow-ups of the 1982 and 1993 Pelotas birth cohorts received approval from the Research Ethics Committee of the School of Medicine of the Federal University of Pelotas. In the 1982 Pelotas birth cohort, follow-ups at 22 years and 30 years of age were approved, respectively, by certificates n. 029/2003 and n. 16/12. The follow-up at 22 years of age in the 1993 Pelotas birth cohort was approved under certificate n. 1,250,366.

## Results

In the 1982 cohort, 4,296 and 3,635 individuals were analyzed at 22 and 30 years of age, respectively. In the 1993 cohort, 3,744 individuals were analyzed at 22 years of age (Table 1). In all follow-ups, more than two-thirds had self-declared White skin color and less than 10% had up to four years of schooling.

**Table 1:** Description of sample according to sociodemographic and metabolic characteristics of 1993 (22 years) and 1982 (22 and 30 years) Pelotas (Brazil) birth cohorts.

Characteristics	1993 cohort	1982 cohort	
	22 years (n = 3,744)	22 years (n = 4,296)	30 years (n = 3,635)
	n (%)	n (%)	n (%)
Sex			
Male	1,778 (47.5)	2,213 (51.5)	1,751 (48.2)
Female	1,966 (52.5)	2,083 (48.5)	1,882 (51.8)
Skin color			
White	2.234 (66.1)	3,238 (78.1)	2,817 (78.6)
Black	528 (15.6)	673 (16.2)	580 (16.2)
Mixed-race	617 (19.3)	235 (5.7)	188 (5.2)
Schooling (years of study)			
0-4	109 (2.9)	349 (8.1)	223 (6.1)
5-8	984 (26.3)	1,208 (28.1)	724 (19.9)
9-11	1,534 (41.0)	2,070 (48.2)	1,092 (30.1)
≥ 12	1,117 (29.8)	669 (15.6)	1,596 (43.9)
Low HDL * ** ***			
No	2.160 (61.9)	3,088 (80.8)	3,069 (86.7)
Yes	1.327 (38.1)	736 (19.2)	472 (13.3)
Blood glucose ≥ 100mg/dL * **			
No	2,746 (79.0)	2,375 (63.6)	2,911 (82.9)
Yes	732 (21.0)	1,357 (36.4)	600 (17.1)
Triglycerides ≥ 150mg/dL * **			
No	3,026 (87.0)	3,159 (82.6)	2,740 (77.4)
Yes	450 (13.0)	665 (17.4)	801 (22.6)
Central obesity ^#^ (IDF)			
No	2,581 (72.0)	3,389 (79.1)	2,020 (56.6)
Yes	1,006 (28.0)	897 (20.9)	1.547 (43.4)
Central obesity ^#^ (NCEP-ATP III)			
No	3,174 (88.5)	3,936 (91.8)	2,911 (81.6)
Yes	413 (11.5)	350 (8.2)	656 (18.4)
SBP ≥ 130mmHg or DBP ≥ 85mmHg *			
No	2,212 (61.6)	3,226 (75.2)	2,584 (71.3)
Yes	1,381 (38.4)	1,065 (24.8)	1,040 (28.7)
Meabolic syndrome (IDF) ^##^			
No	2,823 (73.4)	3,274 (88.7)	2,949 (84.5)
Yes	627 (18.2)	416 (11.3)	542 (15.5)
Metabolic syndrome (NCEP-ATP III) ^##^			
No	2,984 (86.5)	3,373 (91.4)	3,117 (89.3)
Yes	466 (13.5)	317 (8.6)	374 (10.7)

DBP: diastolic blood pressure; IDF: International Diabetes Federation; NCEP-ATP III: *National Cholesterol Education Program Adult Treatment Panel III*; SBP: sistolyc blood pressure.

* Or in treatment;

** Random;

*** HDL cholesterol (low when ˂ 40mg/dL for men and ˂ 50mg/dL for women);

^#^ Measured by waist circumference (present when - IDF: ≥ 90/80cm and NCEP-ATP III: 102/89cm for men/women);

^##^ Diagnosis of metabolic syndrome by presence of three or more metabolic risk factors using IDF and NCEP-ATP III criteria. Characteristics with smaller analytical sample in follow-ups of 1993 cohort (22 years, n = 3,450) and 1982 cohort (22 years, n = 3,690 and 30 years, n = 3,491).

High blood glucose was found in a higher proportion (36.4%) at 22 years of age in the 1982 cohort. The highest rates of low HDL cholesterol (38.1%) and high blood pressure (38.4%) were found at 22 years of age in the 1993 cohort (Table 1)

The other metabolic alterations analyzed were more frequent at 30 years of age (1982 cohort): high triglycerides (22.6%) and central obesity (43.4% using the IDF criteria and 18.4% using the NCEP-ATP III criteria) (Table 1).

The prevalence of metabolic syndrome varied according to the criteria used and was consistently higher by the IDF compared to the NCEP-ATP III in both cohorts and at both ages analyzed. In the 1982 cohort, the prevalence increased from 22 to 30 years of age (11.3% to 15.5% by the IDF and 8.6% to 10.7% by the NCEP-ATP III). In the 1993 cohort, the prevalence at 22 years of age was 18.2% (IDF) and 13.5% (NCEP-ATP III) (Table 1). 

In the analysis of the prevalence of metabolic alterations by sex, most characteristics differed between men and women at the three follow-ups, except for central obesity at 30 years of age (1982 cohort) and high triglycerides at 22 years of age in the 1993 cohort (Table 2). Women had a higher proportion of low HDL cholesterol, especially at 22 years of age in the 1993 cohort, when the absolute difference between sexes was 14.6 percentage points (p.p.) (95%CI: 11.5; 17.8; p-value < 0.001) (45% for women vs. 30.4% for men). Women also had a greater proportion of central obesity at all follow-ups. The largest absolute difference was found at 22 years of age in the 1993 cohort (10.8p.p.; 95%CI: 7.9; 13,7; p-value < 0.001) using the IDF criteria (Table 2).

**Table 2:** Altered metabolic characteristics according to *National Cholesterol Education Program Adult Treatment Panel III* (NCEP-ATP III) and International Diabetes Federation (IDF) criteria by sex and skin color in 1993 (22 years) and 1982 (22 years and 30 years) Pelotas (Brazil) birth cohorts.

	Low HDL cholesterol *	Triglycerides ≥ 150mg/dL	Blood glucose ≥ 100mg/dL **	High blood pressure ***	Central obesity (NCEP-ATP III) ^#^	Central obesity (IDF) ^##^
		1993 cohort (22 years)					
	n = 3,487	n = 3,484	n = 3,478	n = 3,593	n = 3,587	n = 3,587
	% (95%CI)	% (95%CI)	% (95%CI)	% (95%CI)	% (95%CI)	% (95%CI)
Total	38.1 (36.5; 39.7)	18.1 (16.9; 19.4)	21.0 (19.7; 22.4)	38.4 (36.9; 40.0)	11.5 (10.5; 12.6)	28.0 (26.6; 29.5)
Women	45.0 (42.8; 47.3)	17.8 (16.0; 19.6)	19.5 (17.7; 21.4)	20.8 (19.0; 22.7)	15.7 (14.2; 17.5)	33.2 (31.1; 35.3)
White	44.5 (41.6; 47.5)	22.0 (19.7; 24.6) ^###^	20.6 (18.3; 23.0)	19.2 (17.0; 21.6) ^§^	14.4 (12.5; 16.6)	30.3 (27.7; 33.0) ^###^
Black/Mixed-race	48.2 (44.2; 52.3)	10.8 (8.5; 13.6)	17.5 (14.6; 20.8)	23.4 (20.2; 27.0)	17.9 (15.0; 21.2)	39.0 (35.1; 42.9)
Men	30.4 (28.2; 32.6) ^###^	18.5 (16.7; 20.4)	22.7 (20.8; 24.8) ^§^	57.8 (55.4; 60.0) ^###^	6.9 (5.4; 8.6) ^###^	22.4 (20.5; 24.4) ^###^
White	32.7 (29.9; 35.7) ^§^	19.7 (17.4; 22.3)	22.3 (19.8; 24.9)	58.9 (55.9; 61.9)	6.7 (5.4; 8.5)	23.8 (21.3; 26.6)
Black/Mixed-race	25.8 (22.2; 29.9)	17.6 (14.5; 21.2)	22.9 (19.5; 26.8)	56.0 (51.7; 60.3)	7.7 (5.6; 10.3)	22.6 (19.2; 26.4)
	1982 cohort (22 years)					
	n = 3,824	n = 3,824	n = 3,732	n = 4,291	n = 4,286	n = 4,286
	% (95%CI)	% (95%CI)	% (95%CI)	% (95%CI)	% (95%CI)	% (95%CI)
Total	19.2 (18.0; 20.5)	17.4 (16.2; 18.6)	36.4 (34.8; 37.9)	24.8 (23.5; 26.1)	8.2 (7.4; 9.0)	20.9 (19.7; 22.2)
Women	25.5 (23.6; 27.5)	11.8 (10.4; 13.3)	29.6 (27.6; 31.7)	13.4 (12.0; 15.0)	12.1 (10.7; 13.5)	26.9 (25.0; 28.8)
White	25.1 (22.9; 27.4)	13.1 (11.5; 15.0) ^###^	29.3 (27.0; 31.8)	13.1 (11.5; 14.9)	10.4 (9.0; 12.0) ^###^	24.5 (22.4; 26.6) ^###^
Black/Mixed-race	26.8 (22.7; 31.3)	7.8 (5.6; 10.8)	30.9 (26.5; 35.5)	15.3 (12.2; 19.0)	17.4 (14.1; 21.2)	35.0 (30.7; 39.6)
Men	13.0 (11.6; 14.6) ^###^	22.9 (21.1; 24.9) ^###^	43.2 (40.9; 45.4) ^###^	35.6 (33.6; 37.6) ^###^	4.5 (3.7; 5.4) ^###^	15.3 (13.9; 16.9) ^###^
White	13.2 (11.5; 15.0)	24.6 (22.4; 26.9) ^§^	42.7 (40.2; 45.4)	34.3 (32.0; 36.6) ^§^	4.5 (3.6; 5.6)	16.0 (14.3; 17.9) ^§^
Black/Mixed-race	12.4 (9.5; 15.9)	16.4 (13.1; 20.3)	42.8 (38.0; 47.7)	39.4 (35.0; 43.9)	3.6 (2.3; 5.8)	11.5 (8.9; 14.8)
	1982 cohort (30 years)					
	n = 3,462	n = 3,541	n = 3,511	n = 3,624	n = 3,567	n = 3,567
	% (95%CI)	% (95%CI)	% (95%CI)	% (95%CI)	% (95%CI)	% (95%CI)
Total	13.3 (12.2; 14.5)	22.6 (21.3; 24.0)	17.1 (15.9; 18.4)	28.7 (27.2; 30.2)	18.4 (17.2; 19.7)	43.4 (41.8; 45.0)
Women	15.7 (14.1; 17.5)	14.5 (13.0; 16.3)	12.5 (11.0; 14.1)	14.1 (12.5; 15.7)	22.4 (20.5; 24.4)	44.6 (42.3; 46.9)
White	16.2 (14.3; 18.2)	16.0 (14.2; 18.1) ^###^	13.2 (11.5; 15.1)	12.7 (11.1; 14.5) ^###^	20.3 (18.2; 22.5) ^###^	42.9 (40.3; 45.6) ^§^
Black/Mixed-race	13.9 (10.7; 17.8)	10.1 (7.4; 13.6)	9.8 (7.2; 13.3)	19.5 (15.8; 23.8)	29. 2(24.8; 34.1)	49.7 (44.6; 54.9)
Men	10.9 (9.5; 12.4) ^###^	30.9 (28.8; 33.1) ^###^	21.8 (19.9; 23.8) ^###^	44.0 (41.7; 46.3) ^###^	14.3 (12.8; 16.0) ^###^	42.1 (39.8; 44.4)
White	11.0 (9.4; 12.8)	33.0 (30.5; 35.6) ^§^	21.3 (19.2; 23.6)	43.1 (40.4; 45.7)	15.0 (13.2; 17.0) ^§^	43.8 (41.2; 46.5) ^###^
Black/Mixed-race	10.4 (7.7; 14.0)	24.3 (20.2; 29.0)	22.8 (18.9; 27.4)	47.5 (42.6; 52.5)	10.1 (7.4; 13.5)	34.1 (29.5; 39.0)

95%CI: 95% confidence interval.

* Low HDL cholesterol in women ≤ 50mg/dL and in men ≤ 40mg/dL;

** Measured at random;

*** High when systolic blood pressure ≥ 135mmHg and/or diastolic blood pressure ≥ 85mmHg;

^#^ Central obesity considering high waist circumference ≥ 89cm for women and ≥ 102cm for men;

^##^ Central obesity considering high waist circumference ≥ 80cm for women and ≥ 90cm for men;

^###^ p-value < 0.001;

^§^ p-value < 0.05.

Men had higher blood pressure levels in all follow-ups. The highest proportion was observed at 22 years of age in the 1993 cohort: 57.8% of men vs. 20.8% of women, with an absolute difference of 37.0p.p. (95%CI: 33.9; 39.9; p < 0.001) (Table 2). 

When the sample was stratified by sex and skin color, the prevalence of central obesity (IDF) was higher in Black/Mixed-race women in all cohorts, with significant differences in relation to White women: 8.7p.p. (p-value < 0.001) at 22 years in the 1993 cohort, 10.5p.p. (p-value < 0.001) at 22 years in the 1982 cohort, and 6.8p.p. (p-value < 0.05) at 30 years (1982 cohort). Considering men in the 1982 cohort, the prevalence was higher among Whites, with differences of 4.5p.p. (p-value < 0.05) at 22 years of age and 9.7p.p. (p-value < 0.001) at 30 years of age.

The prevalence of metabolic syndrome was higher among men at all follow-ups. At 30 years of age (1982 cohort), greater absolute and relative inequality between the sexes occurred according to the IDF criteria, with the prevalence more than twofold higher in men compared to women (22% vs. 9.2%) (Table 3). The prevalence of metabolic syndrome was the closest between the sexes at 22 years of age in the 1993 cohort (14.6% for men vs. 12.5% for women) according to the NCEP-ATP III criteria (Table 3).

**Table 3:** Prevalence of metabolic syndrome: absolute difference and prevalence ratio according to sex in 1982 and 1993 (Pelotas) birth cohorts.

	Men	Women	Absolute difference (p.p.)	Prevalence ratio (95%CI) *	p-value *
	% (IC95%)	% (IC95%)			
IDF					
1993 cohort					
22 years	19.7 (17.8; 21.6)	16.8 (15.0; 18.5)	2.9	1.17 (1.02; 1.36)	0.024
1982 cohort					
22 years	13.7 (12.1; 15.3)	8.8 (7.6; 10.1)	4.9	1.56 (1.29; 1.87)	< 0.001
30 years	22.0 (20.0; 23.9)	9.2 (7.8; 10.6)	12.8	2.39 (2.00; 2.82)	< 0.001
NCEP-ATP III					
1993 cohort					
22 years	14.6 (12.8; 16.3)	12.5 (11.0; 14.1)	2.1	1.16 (0.98; 1.37)	0.083
1982 cohort					
22 years	10.7 (9.3; 12.1)	6.5 (5.4; 7.6)	4.2	1.65 (1.33; 2.60)	< 0.001
30 years	15.0 (13.3; 16.7)	6.5 (5.4; 7.7)	8.5	2.30 (1.87; 2.84)	< 0.001

95%CI: 95% confidence interval; IDF: International Diabetes Federation; NCEP-ATP III: *National Cholesterol Education Program Adult Treatment Panel III.*

* Poisson regression with robust variance.

**Figure 1:**
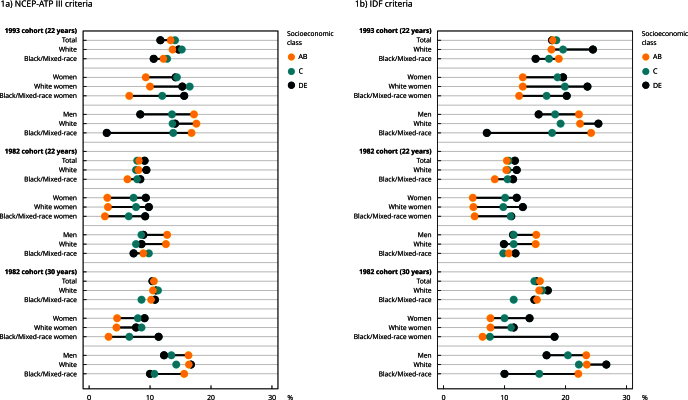
Equiplot with prevalence of metabolic syndrome according to socioeconomic status using classification of Brazilian Association of Research Companies (ABEP) categorized into AB, C, and DE ^36^, sex, and skin color.

Socioeconomic inequalities were identified in the occurrence of metabolic syndrome in relation to sex and skin color according to the Equiplot. In all follow-ups, the prevalence of metabolic syndrome was higher among women with a lower socioeconomic status. In contrast, the prevalence was higher among men with a higher socioeconomic status (Figure 1). Moreover, when the sample was stratified by skin color, greater inequality was observed in the prevalence of metabolic syndrome according to socioeconomic status among Black/Mixed-race individuals. This difference was demonstrated by the greater distance between the points of prevalence of the highest and lowest socioeconomic strata, especially among Black/Mixed-race men in the 1993 cohort and among men and women at 30 years of age in the 1982 cohort (Figure 1).

IDF: International Diabetes Federation; NCEP-ATP III: *National Cholesterol Education Program Adult Treatment Panel III.*

Measures of absolute inequality (SII) and relative inequality (CIX) confirmed this pattern (Table 4). At 22 years of age in the 1982 cohort, women with a lower socioeconomic status had an SII of -8.7p.p. and CIX of -20.1%, in contrast to men, who had an SII of 6.5p.p. and CIX of 7.9%. This pattern was maintained at 30 years of age (1982 cohort), as women had an SII of -6.7p.p. and CIX of -14.8% and men had an SII of 5.7p.p. and CIX of 5.1 (Table 4). Similar patterns were identified in the 1993 cohort at 22 years of age, with higher frequencies of metabolic syndrome found among women with a lower socioeconomic status (SII of -8.5p.p. and CIX of -12.9%) and men with a higher socioeconomic status (SII of 9.6p.p. and CIX of 7.3%) (NCEP-ATP III criteria) (Table 4).

**Table 4:** Absolute and relative inequalities in prevalence of metabolic syndrome according to socioeconomic status assessed using slope index of inequality (SII) and concentration index (CIX) considering *National Cholesterol Education Program Adult Treatment Panel III* (NCEP-ATP III) and International Diabetes Federation (IDF) criteria in 1982 and1993 Pelotas (Brazil) birth cohorts.

	IDF		NCEP-ATP III	
	SII (p.p.) (95%CI)	CIX (95%CI)	SII (p.p.) (95%CI)	CIX (95%CI)
	1993 cohort (22 years)			
Total	-0.8 (-5.7; 4.0)	-1.9 (-6.0; 2.2)	0.5 (-3.8; 4.7)	-2.0 (-6.8; 2.8)
White	-5.8 (-12.3; 0.7)	0.2 (-0.4; 0.8)	-2.5 (-8.2; 3.3)	0.4 (-0.2; 1.1)
Black/Mixed-race	4.6 (-4.0; 13.2)	0.3 (-0.7; 1.4)	1.5 (-5.9; 8.8)	0.2 (-0.9; 1.2)
Women	-10.3 (-16.7; -3.8) **	-9.4 (-0.3; -3.5) **	-8.5 (-14.2; -2.8) **	-12.9 (-19.6; -6.2) ***
White	-14.3 (-22.9; -5.7) **	-11.5 (-19.0; -4.1) **	-11.5 (-19.2; -3.7) **	-11.8 (-20.2; -3.5) **
Black/Mixed-race	-9.6 (-21.2; 2.1)	-5.7 (-16.1; 4.8)	-11.1 (-21.1; -11.7) **	-13.3 (-25.7; -0.9) **
Men	8.6 (1.4; 15.9) **	4.2 (-1.4; 9.8)	9.6 (3.2; 15.9) **	7.3 (0.9; 13.8) **
White	2.8 (-7.1; 12.8)	0.0 (-6.8; 6.9)	7.0 (-1.8; 15.7)	4.2 (-3.8; 12.2)
Black/Mixed-race	19.3 (7.1; 31.6) **	13.1 (2.6; 23.5) **	14.4 (4.0; 24.8) **	13.5 (1.3; 25.6) **
	1982 cohort (22 years)			
Total	-1.8 (-5.9; 2.3)	1.3 (-4.0; 6.6)	-1.1 (-4.8; 2.6)	1.5 (-4.7; 7.8)
White	-2.0 (-6.8; 2.8)	0.6 (-0.2; 1.4)	-1.2 (-5.5; 3.1)	0.6 (-0.1; 1.4)
Black/Mixed-race	-3.4 (-12.2; 5.5)	-0.8 (-2.6; 0.9)	-2.2 (-10.0; 5.5)	-0.8 (-2.5; 1.0)
Women	6.2 ( -0.7; 13.1)	-16.4 (-24.7; -8.2) ***	-8.7 (-13.2; -4.2) ***	-20.1 (-29.7; -10.5) ***
White	7.9 (-0.2; 15.9)	-22.1 (-31.4; -12.7) ***	-9.6 (-14.8; -4.3) ***	-24.2 (-0.35; -13.5) ***
Black/Mixed-race	-2.3 (-15.9; 11.4)	-0.3 (-16.8; 16.2)	-7.7 (-18.1; 2.7)	-7.3 (-28.0; 13.3)
Men	-9.8 (-14.8; -4.7) ***	7.0 (0.6; 13.5) **	6.5 (0.1; 12.9) **	7.9 (0.6; 15.3) *
White	11.4 (17.4; 5.4) ***	9.3 (1.9; 16.8) **	7.9 (3.1; 15.4) **	9.7 (1.1; 18.3) *
Black/Mixed-race	-4.4 (-15.9; 7.2)	1.6 (-14.0; 17.1)	3.2 (-8.5; 14.8)	6.1 (-10.6; 22.7)
	1982 cohort (30 years)			
Total	15.5 (-3.2; 6.3)	1.0 (-3.6; 5.5)	0.1 (-3.9; 4.1)	0.6 (-5.0; 6.2)
White	-1.2 (-6.8; 4.3)	0.2 (-0.3; 0.7)	-1.4 (-6.1; 3.3)	0.2 (-0.3; 0.7)
Black/Mixed-race	3.7 (-6.4; 13.8)	-0.2 (-1.4; 1.0)	1.0 (-7.7; 9.7)	-0.3 (-1.5; 9.4)
Women	-5.8 (-11.1; -0.5) **	-10.5 (-19.4; -1.6) **	-6.7 (-11.2; -2.2) *	-14.8 (-25.5; -4.2) **
White	-6.5 (-12.5; -0.4) **	-11.6 (-21.4; -1.8) **	7.4 (-12.6; -2.2) *	-17.3 (-29.0; -5.2) **
Black/Mixed-race	-11.3 (-25.0; 2.4)	-17.6 (-38.8; 3.6)	-9.7 (-20.8; 1.3)	-19.1 (-42.5; 4.4)
Men	6.7 (-1.0; 14.5)	3.5 (-1.6; 8.6)	5.7 (-1.0; 12.5)	5.1 (-1.2; 11.4)
White	1.6 (-0.8; 11.0)	2.1 (-3.5; 7.8)	3.7 (-4.7; 11.6)	3.9 (-3.1; 10.9)
Black/Mixed-race	1.4 (-0.8; 29.3)	10.6 (-2.4; 23.5)	8.8 (-4.8; 22.3)	14.6 (-1.4; 30.6)

95%CI: 95% confidence interval.

* Classification of socioeconomic status according to Brazilian Association of Research Companies (ABEP) categorized at AB, C, and DE ^36^;

** p-value < 0.05;

*** p-value < 0.001.

The analysis of absolute (SII) and relative (CIX) differences by skin color revealed that the prevalence of metabolic syndrome was higher among women with White skin color and a lower socioeconomic status, with this difference more pronounced at 22 years of age in the 1993 cohort according to the IDF criteria (SII = -14.3p.p.; CIX = -11.5%). Among men, the pattern varied according to the cohort. At 22 years of age, the prevalence of metabolic syndrome was higher among Whites with a higher socioeconomic status in the 1982 cohort (SII = 11.4p.p.; CIX = 9.3%) (Table 4), whereas the prevalence was higher among Black/Mixed-race men with a higher socioeconomic status in the 1993 cohort (22 years of age) (SII = 19.3p.p.; CIX = 13.1%). 

## Discussion

The prevalence of metabolic syndrome was consistently higher when adopting the IDF criteria. In the 1993 cohort, metabolic syndrome already affected 18.2% of the participants at 22 years of age, which is higher than the rates in the 1982 cohort (11.3% at 22 years of age and 15.5% at 30 years of age). The prevalence was also higher among men at all follow-ups. The metabolic alteration with the greatest absolute difference (37p.p.) between the sexes was high blood pressure at 22 years of age in the 1993 cohort (57.8% among men vs. 20.8% among women). When stratified by sex, the NCEP-ATP III criteria revealed greater inequality by socioeconomic status at 22 years of age in the 1982 cohort, as the most affected were women with low socioeconomic status and men with high socioeconomic status (women: SII = -8.7p.p. and CIX = -20.1%; men: SII = 6.5p.p. and CIX = 7.9%). When stratified by sex and skin color, the highest prevalence was found among men 22 years of age with a higher socioeconomic status (Blacks/Mixed-races in the 1993 cohort and Whites in the 1982 cohort). Among women, the pattern was reversed in all follow-ups, with the highest prevalence concentrated in those with White skin color and lower socioeconomic status.

The prevalence of metabolic syndrome increases with age ^19^ ^,^ ^39^ ^,^ ^40^, which was confirmed by the findings of the 1982 cohort. However, the higher prevalence found in the 1993 cohort compared to the 1982 cohort at the same age suggests a possible cohort effect due to different exposures experienced by groups born in different periods - such as dietary changes, urbanization, levels of physical activity, and access to health services ^41^. These findings should be interpreted in the light of the nutritional and epidemiological transitions that have occurred in Brazil, which have accelerated the onset of cardiometabolic diseases ^24^ ^,^ ^41^. Moreover, the differences between cohorts reflect changes in the Brazilian healthcare system. While the participants in the 1982 cohort reached adulthood before the consolidation of the universal healthcare system, those in the 1993 cohort benefited from the expansion of primary care and preventive actions, although access to and the quality of services vary depending on socioeconomic status and the social context ^24^ ^,^ ^25^ ^,^ ^34^ ^,^ ^35^ ^,^ ^42^ ^,^ ^43^.

The prevalence of metabolic syndrome was higher among men. This difference that may be attributed to biological and behavioral mechanisms ^40^ ^,^ ^44^ ^,^ ^45^. Biologically, men tend to accumulate visceral fat, while women accumulate more subcutaneous fat, influenced by testosterone and estrogen, respectively ^40^. Moreover, men and women have different lifestyle habits. Men have higher tobacco consumption ^46^ as well as a diet with excessive amounts of salt and fat ^45^. In turn, women have higher intakes of fruits and vegetables, which may have contributed to this disparity ^44^ ^,^ ^45^ ^,^ ^47^. Excessive salt intake by men may explain the difference in high blood pressure rates between the sexes, as table salt contributes to the increase in blood pressure due to its excess sodium ^48^.

The literature offers conflicting results on the association between sex and metabolic syndrome ^19^ ^,^ ^39^ ^,^ ^49^ ^,^ ^50^. Studies conducted in high-income countries have shown that the prevalence of metabolic syndrome is higher among men with a more favorable socioeconomic status, whereas the condition is more prevalent among women with a less favorable socioeconomic status, especially Black women and those belonging to minority ethnic groups ^19^. Our findings are partially in line with the results reported for these countries. We found that the prevalence of metabolic syndrome was higher among men with a higher socioeconomic status and among women with a lower socioeconomic status. This pattern may be, at least in part, a reflection of behavioral differences. Men from higher social strata in our culture tend to have a more sedentary lifestyle, with less demand for physical activity, especially in the work environment ^27^ ^,^ ^51^, and greater consumption of fats, salt, and ultra-processed foods ^52^. 

Among women, the highest prevalence of metabolic syndrome was among those with a lower socioeconomic status. Such individuals tend to adopt less healthy behaviors, with greater frequencies of smoking, excessive alcohol consumption, a diet low in healthy foods, and less physical activity during leisure time, which are factors that favor the development of metabolic syndrome ^22^ ^,^ ^53^. Moreover, individuals with a low socioeconomic status have unequal access to healthcare services, whereas individuals with higher incomes are more likely to receive better quality care, which may also impact the prevalence of metabolic syndrome ^23^ ^,^ ^54^. An unexpected result was the higher prevalence of metabolic syndrome among White women with a low socioeconomic status. This may be attributed to confounding factors not measured in this descriptive study, such as place of residence or access to inclusive policies, or even to self-declared skin color. The change in racial identification observed in the most recent censuses, with more individuals self-declared as Black/Mixed-race, suggests that the previous prevalence of White skin color may have been overestimated ^30^. Moreover, although low-income Black and Mixed-race individuals face considerable challenges, it is possible that access to social support networks and inclusive policies in some contexts may attenuate, even if only partially, the negative impacts of a low socioeconomic status on health ^42^. 

Another surprising result was the higher prevalence of metabolic syndrome among Black/Mixed-race men with a higher socioeconomic status at 22 years of age in the 1993 cohort. This finding aligns with evidence that Black individuals, even after social ascension, do not always experience the same gains in health and quality of life as White people in the same socioeconomic position ^55^ and may, in some cases, experience a worsening of physical and mental health indicators ^56^.

Social inequalities are central determinants of health operating through structural and cultural mechanisms that increase vulnerability to health problems, such as metabolic syndrome ^25^. In addition to biological and behavioral factors, unexpected patterns of metabolic syndrome may result from the interaction between structural social determinants that shape living conditions and access to resources ^24^ ^,^ ^57^, processes of embodiment that translate these inequalities into biological changes over time ^28^, and intersectional positions that unevenly modulate exposures and vulnerabilities in the population ^58^. The social determinants analyzed in the present study (socioeconomic status, skin color, and sex) have widely described patterns, with greater vulnerability among individuals with a lower income and lower levels of schooling due to greater exposure to environmental, dietary, and occupational risk factors, in addition to less access to healthcare services ^24^. Both skin color and sex define experiences of discrimination, a hierarchy of opportunities, the structure of social roles, and employment trajectories, imposing different workloads and income ^58^. From the eco-social standpoint ^28^, such inequalities become embodied throughout life, that is, social and environmental inequalities are literally incorporated by the body and expressed in biological and metabolic differences, which could explain the higher prevalence of metabolic syndrome in Black/Mixed-race men with the highest socioeconomic status at 22 years of age in the 1993 cohort. These intersections between skin color, socioeconomic status, and sex produce different exposures and specific vulnerabilities that are manifested biologically in distinct metabolic profiles among social groups, such as those observed in the present study.

The formulation of effective public policies requires confronting the social determinants that sustain health inequalities and avoiding placing the emphasis on “individual choices”. Strategies aimed at diminishing the prevalence of metabolic syndrome should consider the reduction in socioeconomic, racial, and gender inequities as well as the creation of social conditions that enable healthy, equitable choices.

This study has some limitations that should be considered. The first refers to losses to follow-up, which are common in cohort studies. The individuals followed up corresponded to about 70% of the initial cohort, which can be considered satisfactory for population-based studies. There was a difference by sex, with greater follow-up of women. As the analyses were stratified by skin color and sex, this bias tends to be minimized. It is also necessary to emphasize that the collections analyzed occurred in the years 2004, 2012, and 2015 and, therefore, the rates described may not fully reflect current levels of metabolic syndrome in the population.

The second limitation regards the measurement of blood pressure at 22 years of age in the 1982 cohort, which was performed with a digital device, differing from the method employed in the other two follow-ups. Although measurements by this type of apparatus are comparable to those of a mercury device, they tend to be slightly higher ^59^. Thus, it is possible that the occurrence of high blood pressure may have been overestimated in this follow-up. The third limitation regards the collection of serum laboratory tests and capillary glucose, which was performed at random and may have overestimated blood glucose levels and, to a lesser extent, triglycerides and HDL, as fasting is not mandatory for the determination of the lipid profile ^60^. 

Lastly, at 22 years of age in the 1982 cohort, capillary blood glucose was measured using the Accu-Chek Advantage (Roche, https://www.roche.com.br/) device, which, despite having good sensitivity (93.8%) and specificity (83.6%) ^61^, tends to overestimate blood glucose ^62^. This may have artificially increased the prevalence of metabolic syndrome in this group. As this was the follow-up with the lowest overall prevalence, the actual difference between the two cohorts at 22 years of age is likely to be even greater than that observed.

In the 1993 cohort (22 years of age), the definition of diabetes, hypertension, and dyslipidemia included self-reported medical diagnoses and the use of medications, which did not occur in the other follow-ups. The level of agreement between the two definitions (with and without self-reports) was perfect (kappa = 1.0) for the three conditions, indicating that the inclusion of self-reported information did not alter the prevalence of these conditions or introduce bias into the analyses. 

The standardization of the collection of the vast majority of data and methodological rigor in the treatment of the information are strengths of the study. Other strengths were the intersectional approach to inequalities and the use of different indicators to assess inequalities both relatively and absolutely, which showed a consistent pattern of inequalities in all follow-ups analyzed. The use of two cohorts for analysis also contributes to the consistency and robustness of the results.

In this study, different patterns of metabolic syndrome were identified according to socioeconomic position, sex, and skin color. The prevalence of metabolic syndrome was higher in men from higher social strata, mainly Black/Mixed-race individuals, and in women from lower social strata, especially those with White skin color. Further studies should be conducted to broaden the understanding of the processes and mechanisms involved in inequalities in the occurrence of metabolic syndrome in the Brazilian population.

## Data Availability

The sources of information used in the study are indicated in the body of the article.
