# Supplementary material for: Occurrence of metabolic syndrome in young adults and inequalities according to sex, skin color, and socioeconomic status: evidence from two birth cohorts in Southern Brazil
Source: Cad Saude Publica. 2026 Apr 27;42:e00178025. doi: 10.1590/0102-311XEN178025 (PMC13170111; doi:10.1590/0102-311XEN178025)
Supplement: Translation

# Translation: Ocorrência de síndrome metabólica em adultos jovens e desigualdades segundo sexo, cor da pele e posição socioeconômica: evidências de duas coortes de nascimentos no Sul do Brasil

**DOI:** 10.1590/0102-311XPT178025

**Authors:** Ana Paula Oliveira Rosses, Gabriela Ávila Marques, Bruna Gonçalves-Silva, Ana M. B. Menezes, Helen Gonçalves, Fernando Hartwig, Bernardo L. Horta, Fernando C. Wehrmeister

**Affiliations:** 1 Programa de Pós-graduação em Epidemiologia, Universidade Federal de Pelotas, Pelotas, Brasil.

**Keywords:** Síndrome Metabólica, Fatores de Risco Cardiometabólico, Desigualdades em Saúde, Estudos de Coortes

## Abstract

Este estudo buscou identificar a prevalência de síndrome metabólica aos 22 e 30 anos abordando desigualdades por sexo, cor da pele e posição socioeconômica, e suas intersecções. Análise transversal utilizando dados das coortes de nascimento de Pelotas, Rio Grande do Sul, Brasil, de 1993 (22 anos) e de 1982 (22 e 30 anos). A síndrome metabólica foi definida pelos critérios da *National Cholesterol Education Program Adult Treatment Panel III* (NCEP-ATP III) e da Federação Internacional de Diabetes (IDF). As desigualdades entre sexo, cor da pele e posição socioeconômica foram descritas em Equiplots e analisadas por medidas absolutas (diferença de prevalência e índice de inclinação da desigualdade – SII]) e relativas (razão de prevalência e índice de concentração – CIX]). Aos 22 anos, a prevalência de síndrome metabólica foi de 18,2% (IDF) e 13,5% (NCEP-ATP III) (coorte de 1993), e de 11,3% (IDF) e 8,6% (NCEP-ATP III) (coorte de 1982). Aos 30 anos (coorte de 1982), foi de 15,5% (IDF) e 10,7% (NCEP-ATP III). A maior desigualdade por posição socioeconômica ocorreu na coorte de 1982 aos 22 anos, pelo NCEP-ATP III: entre mulheres (SII: -8,7p.p.; [IC95%: -13,2; -4,2]; CIX: -20,1%; [IC95%: -29,7; -10,5]) e homens (SII: 6,5p.p.; [IC95%: 0,1; 12,9]; CIX: 7,9%; [IC95%: 0,6; 15,3]). Os subgrupos mais acometidos foram: mulheres brancas de posição socioeconômica inferior, homens pretos/pardos (coorte de 1993) e homens brancos (coorte de 1982) de posição socioeconômica superior. Nossos resultados reforçam que a síndrome metabólica não está distribuída de forma homogênea nesta população e que sua ocorrência pode ser influenciada por intersecções entre posição socioeconômica, sexo e cor da pele.

## Introdução

Doenças cardiometabólicas são a principal causa de mortalidade global desde a década de 1940 e, atualmente são responsáveis por cerca de um terço das mortes no mundo ^1^ ^,^ ^2^ ^,^ ^3^, contribuindo com cerca de 20 milhões de mortes por ano, das quais 80% acontecem em países de média e baixa renda ^4^. As doenças cardiometabólicas têm contribuído progressivamente para o aumento dos custos financeiros com saúde ao longo dos anos, tanto de forma direta, devido às internações, acompanhamentos, procedimentos e tratamentos, quanto de forma indireta, pela perda de produtividade ^5^ ^,^ ^6^ ^,^ ^7^ ^,^ ^8^.

A síndrome metabólica é um conjunto de alterações metabólicas que aumentam o risco de doenças cardiometabólicas. Existem diversos critérios para o diagnóstico de síndrome metabólica, porém os mais utilizados são os da *National Cholesterol Education Program Adult Treatment Panel III* (NCEP-ATP III) e da Federação Internacional de Diabetes (IDF, acrônimo em inglês) ^9^. O diagnóstico de síndrome metabólica é estabelecido, por ambos critérios, quando pelo menos três dos cinco fatores a seguir estão presentes: obesidade central (circunferência abdominal ≥ 102/88cm para homens/mulheres, segundo o NCEP-ATP III), ou 90/80cm pela IDF, pressão arterial elevada (≥ 135/85mmHg), dislipidemia (triglicerídeos ≥ 150mg/dL ou HDL-colesterol < 40mg/dL em homens e < 50mg/dL em mulheres) e glicemia de jejum elevada (≥ 100mg/dL) ou diabetes ^9^.

Estudos demonstraram que a presença da síndrome metabólica praticamente dobra o risco para doença cardiovascular ^10^ ^,^ ^11^ ^,^ ^12^ ^,^ ^13^, sendo também reconhecida como um preditor robusto para o desenvolvimento de diabetes tipo 2, aumentando seu risco em até cinco vezes em adultos ^14^ ^,^ ^15^ ^,^ ^16^. Embora as doenças cardiometabólicas geralmente se manifestem clinicamente na população mais velha, a síndrome metabólica e seus componentes se estabelecem cada vez mais precocemente na vida adulta, com evidências que apontam sua origem na infância e adolescência ^17^.

A síndrome metabólica e as doenças cardiometabólicas têm origem multifatorial, sendo influenciadas por fatores biológicos, comportamentais e socioeconômicos ^18^. A desigualdade social influencia tanto o surgimento quanto à progressão dessas condições, de modo que indivíduos em maior vulnerabilidade, como aqueles de baixa renda, menor escolaridade e pertencentes a minorias raciais ou étnicas, apresentam maior risco de adoecimento ^19^ ^,^ ^20^ ^,^ ^21^. A relação entre posição socioeconômica e o risco de síndrome metabólica e doenças cardiometabólicas difere no cenário global. Em países de alta renda, o padrão é tipicamente linear e bem estabelecido, onde a baixa posição socioeconômica está consistentemente associado ao maior risco de doença cardiometabólica ^1^ ^,^ ^18^ ^,^ ^22^. Contudo, em países de baixa e média renda o padrão é mais complexo devido à transição epidemiológica, onde o maior risco de doença cardiometabólica afeta inicialmente os estratos de melhor posição socioeconômica e tende migrar para a base social com o avanço socioeconômico ^23^ ^,^ ^24^ ^,^ ^25^.

O Brasil, como um país de média renda e historicamente marcado por profundas iniquidades, insere-se nesse contexto de transição epidemiológica ^24^ ^,^ ^26^ ^,^ ^27^. A aplicação direta de achados de países de alta renda ao Brasil é, portanto, limitada, exigindo a compreensão detalhada de como as desigualdades estruturais de cor da pele e sexo interagem com a posição socioeconômica para determinar o risco de síndrome metabólica ^28^. Nesse sentido, o objetivo deste estudo é estimar a prevalência de síndrome metabólica pelos critérios IDF e NCEP-ATP III nos acompanhamentos de 22 e 30 anos nas coortes de nascimentos de Pelotas, Rio Grande do Sul, Brasil, de 1982 e 1993 e analisar as desigualdades de sua ocorrência em relação a sexo, cor da pele e posição socioeconômica.

## Metodologia

### Delineamento e local

Foram realizadas análises transversais utilizando dados das coortes de nascimentos de Pelotas de 1982 e de 1993. Pelotas é um município de médio porte localizado no extremo Sul do Brasil, com uma população de 325.685 habitantes em 2022, dos quais 23,9% se autodeclaram pretos/pardos ^29^ ^,^ ^30^. 

Todas as crianças que tiveram nascimentos hospitalares entre 1º de janeiro e 31 de dezembro nos anos de 1982 e 1993, cujas famílias residiam na zona urbana do Município de Pelotas Rio Grande do Sul fazem parte das coortes. A amostra inicial foi composta por 5.914 indivíduos em 1982 e 5.249 em 1993 ^31^ ^,^ ^32^ ^,^ ^33^ ^,^ ^34^ ^,^ ^35^. 

As análises foram conduzidas com base em três avaliações: aos 22 e 30 anos na coorte de 1982, e aos 22 anos na coorte de 1993. Essa estratégia permitiu investigar mudanças em um mesmo grupo de indivíduos, bem como comparar diferentes gerações em idades semelhantes. 

Os participantes têm sido acompanhados prospectivamente em diferentes fases da vida, com taxas de seguimento de 77,4% aos 22 anos e 68,1% aos 30 anos da coorte de 1982 e 76,3% aos 22 anos da coorte de 1993. As coletas das informações de todos os acompanhamentos são realizadas por equipes treinadas de forma padronizada, tanto para a aplicação dos questionários, quanto para a aferição das medidas antropométricas e demais exames realizados ^31^ ^,^ ^32^ ^,^ ^33^ ^,^ ^34^ ^,^ ^35^. Maiores detalhamentos sobre aspectos metodológicos das coortes foram previamente publicados ^31^ ^,^ ^32^ ^,^ ^33^ ^,^ ^34^ ^,^ ^35^.

### Desfechos

A presença de síndrome metabólica foi avaliada aos 22 e 30 anos na coorte de 1982 e aos 22 anos na coorte de 1993, conforme os critérios do NCEP-ATP III e da IDF. Ambos os critérios diagnósticos consideram cinco componentes - glicemia, colesterol HDL, triglicerídeos, pressão arterial e circunferência abdominal - sendo o diagnóstico de síndrome metabólica estabelecido quando pelo menos três desses cinco parâmetros estão alterados. A principal diferença entre os critérios refere-se ao ponto de corte adotado para a circunferência abdominal, que é mais restritivo no critério da IDF. As formas de coleta, pontos de corte e a operacionalização das variáveis estão detalhadas no Quadro 1.

**Quadro 1:** Variáveis de desfecho e critérios de definição da síndrome metabólica segundo o *National Cholesterol Education Program Adult Treatment Panel III* (NCEP-ATP III) e a Federação Internacional de Diabetes (IDF, acrônimo em inglês) nas coortes de nascimento de Pelotas, Rio Grande do Sul, Brasil, de 1982 (22 e 30 anos) e 1993 (22 anos).

VARIÁVEIS E CRITÉRIOS	COORTE DE 1982		COORTE DE 1993
	22 ANOS	30 ANOS	22 ANOS
Obesidade central	Medida padronizada com fita métrica inextensível. Média de duas aferições no ponto médio entre a última costela e o osso ilíaco. Alterada quando circunferência abdominal ≥ 102/88cm (NCEP-ATP III) e 90/80cm (IDF), para homens/mulheres		
Pressão arterial	Aferida com esfigmomanômetro digital de pulso (Omron HEM-629). Média de duas medidas em repouso de pelo menos cinco minutos no punho direito. Alterada quando ≥ 130/85mmHg, ou uso autorreferido de medicação anti-hipertensiva	Aferida com esfigmomanômetro digital (Omron HEM-705CPINT), em duas medições padronizadas no início e ao final da avaliação antropométrica no braço esquerdo, com participante sentado em repouso e manguito adequado ao perímetro braquial. Alterada se ≥ 130/85mmHg ou em uso de medicação anti-hipertensiva; coorte de 1993 também por diagnóstico médico de hipertensão autorreferido	
Glicemia	Coleta ao acaso. Avaliada por glicemia capilar, utilizando um glicosímetro portátil (Accu-Chek Advantage - Roche). Alterada se ≥ 100mg/dL e/ou uso de medicamento autorreferido para diabetes	Coleta ao acaso por punção venosa. Avaliada por método enzimático colorimétrico utilizando os analisadores Selectra 2 (Merck) e o BS-380 (Shenzhen Mindray Bio-Medical Electronics Co.). Alterada se ≥ 100mg/dL ou uso de medicamento autorreferido para diabetes; coorte de 1993 também por diagnóstico médico de diabetes autorreferido	
Triglicerídeos	Coleta ao acaso por punção venosa. Avaliada por método enzimático colorimétrico com os analisadores Selectra 2 (Merck) e o BS-380 (Shenzhen Mindray Bio-Medical Electronics Co.). Alterado se ≥ 150mg/dL ou uso autorreferido de medicação para dislipidemia; coorte de 1993 também por diagnóstico médico de dislipidemia autorreferido		
HDL colesterol	Coleta ao acaso por punção venosa. Avaliado por método direto ultrassensível aos 22 anos de 1982, nos demais foi utilizado método enzimático colorimétrico. Utilizados os analisadores Selectra 2 (Merck) e o BS-380 (Shenzhen Mindray Bio-Medical Electronics Co.). Alterado se ≤ 40mg/dL em homens ou ≤ 50mg/dL em mulheres ou uso de medicamento para dislipidemia autorreferido; coorte de 1993 também por diagnóstico médico de dislipidemia autorreferido		
Síndrome metabólica NCEP-ATP III	Presença de três ou mais componentes alterados: triglicerídeos ≥ 150mg/dL, HDL < 40mg/dL (homens) ou < 50mg/dL (mulheres), pressão arterial ≥ 130/85mmHg, glicemia ≥ 100mg/dL, e circunferência abdominal ≥ 102cm (homens) ou ≥ 88cm (mulheres)		
Síndrome metabólica IDF	Presença de três ou mais componentes alterados acima diferenciando da anterior apenas na circunferência abdominal ≥ 90cm (homens) ou ≥ 80cm (mulheres)		

### Exposições

A informação sobre sexo (masculino/feminino) dos participantes foi coletada nos estudos perinatais de ambas as coortes. Já a cor da pele foi referida pelos participantes usando as categorias utilizadas pelo Instituto Brasileiro de Geografia e Estatística (IBGE) - aos 22 anos para a coorte de 1982 e aos 15 anos para a coorte de 1993.

A posição socioeconômica foi mensurada utilizando a classificação da Associação Brasileira de Empresas de Pesquisa (ABEP) ^36^ e agrupada em 3 categorias: AB, C e DE. A escolaridade, em anos completos de educação formal, foi categorizada em 0-4, 5-8, 9-11 e ≥ 12 anos de estudo. Ambas as medidas ocorreram no mesmo momento da avaliação da síndrome metabólica para cada coorte: aos 22 e 30 anos na coorte de 1982 e aos 22 anos na coorte de 1993.

### Análise dos dados

Foram excluídos da análise as gestantes e participantes com dados faltantes além de indivíduos de cor da pele amarela ou indígenas devido seu pequeno número. 

Para investigar possíveis interações entre os estratificadores (sexo, cor da pele, posição socioeconômica e escolaridade), foi realizada análise dos termos de interação em modelos de regressão logística em cada coorte, tendo a ocorrência de síndrome metabólica, definida pelos critérios IDF e NCEP-ATP III, como desfecho dicotômico. Não foi encontrada evidência de interação (p < 0,20) entre posição socioeconômica e cor da pele em nenhum dos acompanhamentos analisados. Por outro lado, sexo apresentou interação com os três estratificadores em todos os acompanhamentos. A interação de maior valor de p foi observada entre sexo e cor da pele (valor de p = 0,17) pelo critério NCEP-ATP III aos 22 anos na coorte de 1993, enquanto as demais interações apresentaram valor de p < 0,05.

Todas as análises foram realizadas separadamente para os critérios NCEP-ATP III e IDF. Foram analisadas as medidas de desigualdades absolutas e relativas. Para desigualdade entre os sexos (variável dicotômica) foi calculada a diferença da prevalência de síndrome metabólica e razão de prevalência de acordo com os dois critérios (NCEP-ATP III e IDF). As proporções foram comparadas utilizando o teste do qui-quadrado de Pearson, considerando como uma diferença significativa quando apresentou um valor de p < 0,05. 

Para visualizar e identificar padrões de desigualdade, foram utilizados Equiplots (https://equidade.org/en/equiplot). Nesse tipo de gráfico, cada ponto representa a prevalência do desfecho de interesse em subgrupos populacionais definidos por sexo e cor da pele e a distância entre os pontos expressa a magnitude da desigualdade entre as categorias analisadas ^37^. 

Ainda para a posição socioeconômica (variável ordinal), foi utilizado o índice de inclinação da desigualdade (SII, acrônimo em inglês). Essa medida avalia a diferença absoluta na prevalência de síndrome metabólica ao longo de toda a distribuição ^38^. Para desigualdade relativa, foi utilizado o índice de concentração (CIX, acrônimo em inglês). Esse índice quantifica a concentração de um desfecho de saúde em diferentes pontos da distribuição de renda ou outra medida socioeconômica ^38^. 

As análises foram realizadas utilizando o programa Stata, versão 18.0 (https://www.stata.com), e o Excel, versão 2505 Build 16.0.18827.20102 (https://products.office.com/).

### Aspectos éticos

Os acompanhamentos das coortes de nascimentos de Pelotas de 1982 e 1993 foram aprovados pelo Comitê de Ética em Pesquisa da Faculdade de Medicina, Universidade Federal de Pelotas. Na coorte de nascimentos 1982, os acompanhamentos aos 22 anos e 30 anos foram aprovados, respectivamente, pelos ofícios nº 029/2003 e nº 16/12. O acompanhamento aos 22 anos da coorte de nascimentos de 1993 foi aprovado sob o parecer nº 1.250.366.

## Resultados

Foram avaliados da coorte de 1982 aos 22 e 30 anos 4.296 e 3.635 indivíduos, respectivamente, e 3.744 indivíduos da coorte 1993 aos 22 anos (Tabela 1). Em todos os acompanhamentos, mais de dois terços se declararam brancos e menos de 10% possuíam até quatro anos completos de escolaridade.

**Tabela 1:** Descrição da amostra de acordo com características sociodemográficas e metabólicas das coortes de nascimento de Pelotas, Rio Grande do Sul, Brasil, de 1982 (22 e 30 anos) e 1993 (22 anos).

Características	Coorte de 1993	Coorte de 1982	
	22 anos (n = 3.744)	22 anos (n = 4.296)	30 anos (n = 3.635)
	n (%)	n (%)	n (%)
Sexo			
Masculino	1.778 (47,5)	2.213 (51,5)	1.751 (48,2)
Feminino	1.966 (52,5)	2.083 (48,5)	1.882 (51,8)
Cor da pele			
Branca	2.234 (66,1)	3.238 (78,1)	2.817 (78,6)
Preta	528 (15,6)	673 (16,2)	580 (16,2)
Parda	617 (19,3)	235 (5,7)	188 (5,2)
Escolaridade (anos estudo)			
0-4	109 (2,9)	349 (8,1)	223 (6,1)
5-8	984 (26,3)	1.208 (28,1)	724 (19,9)
9-11	1.534 (41,0)	2.070 (48,2)	1.092 (30,1)
≥ 12	1.117 (29,8)	669 (15,6)	1.596 (43,9)
HDL baixo * ** ***			
Não	2.160 (61,9)	3.088 (80,8)	3.069 (86,7)
Sim	1.327 (38,1)	736 (19,2)	472 (13,3)
Glicemia ≥ 100mg/dL * **			
Não	2.746 (79,0)	2.375 (63,6)	2.911 (82,9)
Sim	732 (21,0)	1.357 (36,4)	600 (17,1)
Triglicerídeos ≥ 150mg/dL * **			
Não	3.026 (87,0)	3.159 (82,6)	2.740 (77,4)
Sim	450 (13,0)	665 (17,4)	801 (22,6)
Obesidade central ^#^ (IDF)			
Não	2.581 (72,0)	3.389 (79,1)	2.020 (56,6)
Sim	1.006 (28,0)	897 (20,9)	1.547 (43,4)
Obesidade central ^#^ (NCEP-ATP III)			
Não	3.174 (88,5)	3.936 (91,8)	2.911 (81,6)
Sim	413 (11,5)	350 (8,2)	656 (18,4)
PAS ≥ 130mmHg ou PAD ≥ 85mmHg *			
Não	2.212 (61,6)	3.226 (75,2)	2.584 (71,3)
Sim	1.381 (38,4)	1.065 (24,8)	1.040 (28,7)
Síndrome metabólica (IDF) ^##^			
Não	2.823 (73,4)	3.274 (88,7)	2.949 (84,5)
Sim	627 (18,2)	416 (11,3)	542 (15,5)
Síndrome metabólica (NCEP-ATP III) ^##^			
Não	2.984 (86,5)	3.373 (91,4)	3.117 (89,3)
Sim	466 (13,5)	317 (8,6)	374 (10,7)

IDF: Federação Internacional de Diabetes; NCEP-ATP III: *National Cholesterol Education Program Adult Treatment Panel III*; PAD: pressão arterial diastólica; PAS: pressão arterial sistólica.

* Ou em tratamento;

** Ao acaso;

*** HDL colesterol (baixo quando ˂ 40mg/dL para homens e ˂ 50mg/dL para mulheres);

^#^ Medida pela circunferência abdominal (presente quando - IDF: ≥ 90/80cm e NCEP-ATP III: 102/89cm para homens/mulheres);

^##^ Diagnóstico de síndrome metabólica pela presença de três ou mais fatores de risco metabólicos utilizando critério da IDF e da NCEP-ATP III. Características com menor amostra analítica nos acompanhamentos coorte de 1993 (22 anos, n = 3.450) e coorte de 1982 (22 anos, n = 3.690) e (30 anos, n = 3.491).

A glicemia elevada foi encontrada em maior proporção (36,4%) aos 22 anos (coorte de 1982). Aos 22 anos (coorte de 1993) foi observada a maior prevalência de HDL colesterol baixo (38,1%) e pressão arterial elevada (38,4%) (Tabela 1).

As demais alterações metabólicas avaliadas foram mais frequentes aos 30 anos (coorte de 1982): triglicerídeos (22,6%), obesidade central (43,4% IDF e 18,4% NCEP-ATP III) (Tabela 1).

As prevalências de síndrome metabólica variaram conforme o critério utilizado, sendo consistentemente maiores pelo IDF em comparação ao NCEP-ATP III em todas as coortes e idades avaliadas. Na coorte de 1982, aumentaram dos 22 para os 30 anos (11,3% para 15,5%, pelo IDF, e 8,6% para 10,7%, pelo NCEP-ATP III). Na coorte de 1993, aos 22 anos, as prevalências foram de 18,2% (IDF) e 13,5% (NCEP-ATP III) (Tabela 1). 

Na avaliação da prevalência das alterações metabólicas por sexo, a maioria das características diferiram entre homens e mulheres nos três acompanhamentos, exceto obesidade central aos 30 anos (coorte de 1982) e triglicerídeos elevados aos 22 anos (coorte de 1993) (Tabela 2). Mulheres apresentaram maior proporção de HDL colesterol baixo, especialmente aos 22 anos da coorte de 1993, quando as mulheres e homens tiveram uma a diferença absoluta de 14,6 pontos percentuais (p.p.) (IC95%: 11,5; 17,8; valor de p < 0,001) (mulheres 45% *vs*. homens 30,4%). As mulheres também apresentaram maior prevalência de obesidade central em todos os acompanhamentos avaliados. Aos 22 anos (coorte de 1993), foi observada a maior diferença absoluta (10,8p.p.; IC95%: 7,9; 13,7; valor de p < 0,001) pelo critério IDF (Tabela 2). 

**Tabela 2:** Alterações metabólicas, segundo critérios da *National Cholesterol Education Program Adult Treatment Panel III* (NCEP-ATP III) e da Federação Internacional de Diabetes (IDF, acrônimo em inglês), por sexo e cor da pele, das coortes de nascimento de Pelotas, Rio Grande do Sul, Brasil, de 1982 (22 e 30 anos) e 1993 (22 anos).

	HDL colesterol baixo *	Triglicerídeos ≥ 150mg/dL	Glicemia ≥ 100mg/dL **	Pressão arterial elevada ***	Obesidade central (NCEP-ATP III) ^#^	Obesidade central (IDF) ^##^
	Coorte de 1993 (22 anos)					
	n = 3.487	n = 3.484	n = 3.478	n = 3.593	n = 3.587	n = 3.587
	% (IC95%)	% (IC95%)	% (IC95%)	% (IC95%)	% (IC95%)	% (IC95%)
Total	38,1 (36,5; 39,7)	18,1 (16,9; 19,4)	21,0 (19,7; 22,4)	38,4 (36,9; 40,0)	11,5 (10,5; 12,6)	28,0 (26,6; 29,5)
Mulheres	45,0 (42,8; 47,3)	17,8 (16,0; 19,6)	19,5 (17,7; 21,4)	20,8 (19,0; 22,7)	15,7 (14,2; 17,5)	33,2 (31,1; 35,3)
Brancas	44,5 (41,6; 47,5)	22,0 (19,7; 24,6) ^###^	20,6 (18,3; 23,0)	19,2 (17,0; 21,6) ^§^	14,4 (12,5; 16,6)	30,3 (27,7; 33,0) ^###^
Pretas/Pardas	48,2 (44,2; 52,3)	10,8 (8,5; 13,6)	17,5 (14,6; 20,8)	23,4 (20,2; 27,0)	17,9 (15,0; 21,2)	39,0 (35,1; 42,9)
Homens	30,4 (28,2; 32,6) ^###^	18,5 (16,7; 20,4)	22,7 (20,8; 24,8) ^§^	57,8 (55,4; 60,0) ^###^	6,9 (5,4; 8,6) ^###^	22,4 (20,5; 24,4) ^###^
Brancos	32,7 (29,9; 35,7) ^§^	19,7 (17,4; 22,3)	22,3 (19,8; 24,9)	58,9 (55,9; 61,9)	6,7 (5,4; 8,5)	23,8 (21,3; 26,6)
Pretos/Pardos	25,8 (22,2; 29,9)	17,6 (14,5; 21,2)	22,9 (19,5; 26,8)	56,0 (51,7; 60,3)	7,7 (5,6; 10,3)	22,6 (19,2; 26,4)
	Coorte de 1982 (22 anos)					
	n = 3.824	n = 3.824	n = 3.732	n = 4.291	n = 4.286	n = 4.286
	% (IC95%)	% (IC95%)	% (IC95%)	% (IC95%)	% (IC95%)	% (IC95%)
Total	19,2 (18,0; 20,5)	17,4 (16,2; 18,6)	36,4 (34,8; 37,9)	24,8 (23,5; 26,1)	8,2 (7,4; 9,0)	20,9 (19,7; 22,2)
Mulheres	25,5 (23,6; 27,5)	11,8 (10,4; 13,3)	29,6 (27,6; 31,7)	13,4 (12,0; 15,0)	12,1 (10,7; 13,5)	26,9 (25,0; 28,8)
Brancas	25,1 (22,9; 27,4)	13,1 (11,5; 15,0) ^###^	29,3 (27,0; 31,8)	13,1 (11,5; 14,9)	10,4 (9,0; 12,0) ^###^	24,5 (22,4; 26,6) ^###^
Pretas/Pardas	26,8 (22,7; 31,3)	7,8 (5,6; 10,8)	30,9 (26,5; 35,5)	15,3 (12,2; 19,0)	17,4 (14,1; 21,2)	35,0 (30,7; 39,6)
Homens	13,0 (11,6; 14,6) ^###^	22,9 (21,1; 24,9) ^###^	43,2 (40,9; 45,4) ^###^	35,6 (33,6; 37,6) ^###^	4,5 (3,7; 5,4) ^###^	15,3 (13,9; 16,9) ^###^
Brancos	13,2 (11,5; 15,0)	24,6 (22,4; 26,9) ^§^	42,7 (40,2; 45,4)	34,3 (32,0; 36,6) ^§^	4,5 (3,6; 5,6)	16,0 (14,3; 17,9) ^§^
Pretos/Pardos	12,4 (9,5; 15,9)	16,4 (13,1; 20,3)	42,8 (38,0; 47,7)	39,4 (35,0; 43,9)	3,6 (2,3; 5,8)	11,5 (8,9; 14,8)
	Coorte de 1982 (30 anos)					
	n = 3.462	n = 3.541	n = 3.511	n = 3.624	n = 3.567	n = 3.567
	% (IC95%)	% (IC95%)	% (IC95%)	% (IC95%)	% (IC95%)	% (IC95%)
Total	13,3 (12,2; 14,5)	22,6 (21,3; 24,0)	17,1 (15,9; 18,4)	28,7 (27,2; 30,2)	18,4 (17,2; 19,7)	43,4 (41,8; 45,0)
Mulheres	15,7 (14,1; 17,5)	14,5 (13,0; 16,3)	12,5 (11,0; 14,1)	14,1 (12,5; 15,7)	22,4 (20,5; 24,4)	44,6 (42,3; 46,9)
Brancas	16,2 (14,3; 18,2)	16,0 (14,2; 18,1) ^###^	13,2 (11,5; 15,1)	12,7 (11,1; 14,5) ^###^	20,3 (18,2; 22,5) ^###^	42,9 (40,3; 45,6) ^§^
Pretas/Pardas	13,9 (10,7; 17,8)	10,1 (7,4; 13,6)	9,8 (7,2; 13,3)	19,5 (15,8; 23,8)	29, 2(24,8; 34,1)	49,7 (44,6; 54,9)
Homens	10,9 (9,5; 12,4) ^###^	30,9 (28,8; 33,1) ^###^	21,8 (19,9; 23,8) ^###^	44,0 (41,7; 46,3) ^###^	14,3 (12,8; 16,0) ^###^	42,1 (39,8; 44,4)
Brancos	11,0 (9,4; 12,8)	33,0 (30,5; 35,6) ^§^	21,3 (19,2; 23,6)	43,1 (40,4; 45,7)	15,0 (13,2; 17,0) ^§^	43,8 (41,2; 46,5) ^###^
Pretos/Pardos	10,4 (7,7; 14,0)	24,3 (20,2; 29,0)	22,8 (18,9; 27,4)	47,5 (42,6; 52,5)	10,1 (7,4; 13,5)	34,1 (29,5; 39,0)

IC95%: intevalo de 95% de confiança.

* HDL colesterol baixo em mulheres ≤ 50mg/dL e em homens ≤ 40mg/dL;

** Medida ao acaso;

*** Pressão arterial elevada quando pressão arterial sistólica ≥ 135mmHg e/ou pressão arterial diastólica ≥ 85mmHg;

^#^ Obesidade central considerando circunferência abdominal elevada em mulheres ≥ 89cm e em homens ≥ 102cm;

^##^ Obesidade central considerando circunferência abdominal elevada em mulheres ≥ 80cm e em homens ≥ 90cm;

^###^ Valor de p < 0,001;

^§^ Valor de p < 0,05.

Em todos os acompanhamentos, os homens apresentaram maiores níveis pressóricos. A maior proporção foi observada aos 22 anos (coorte de 1993), com 57,8% dos homens *vs*. 20,8% das mulheres, com diferença absoluta de 37,0p.p. (IC95%: 33,9; 39,9; valor de p < 0,001) (Tabela 2). 

Quando a amostra foi estratificada por sexo e cor da pele, foi observada maior prevalência de obesidade central (IDF) em mulheres pretas/pardas em todas as coortes, com diferenças significativas em relação às mulheres brancas: 8,7p.p. (p < 0,001) aos 22 anos (coorte de 1993), 10,5p.p. (p < 0,001) aos 22 anos (coorte de 1982) e 6,8p.p. (p < 0,05) aos 30 anos (coorte de 1982). Entre os homens da coorte de 1982, foi observada maior prevalência entre os brancos, com diferenças de 4,5p.p. (p < 0,05) aos 22 anos e 9,7p.p. (p < 0,001) aos 30 anos.

Em todos os acompanhamentos, a prevalência de síndrome metabólica foi maior entre os homens. Aos 30 anos (coorte 1982) pelo critério IDF, houve maior desigualdade absoluta e relativa entre os sexos, sendo a prevalência em homens duas vezes maior entre as mulheres (22% *vs*. 9,2%) (Tabela 3). Já aos 22 anos (coorte de 1993), a prevalência de síndrome metabólica entre os sexos foi a mais próxima (homens 14,6% *vs*. mulheres 12,5% pelo critério NCEP-ATP III) (Tabela 3).

**Tabela 3:** Prevalência de síndrome metabólica, diferença absoluta e razão de prevalência segundo sexo, nas coortes de nascimento de Pelotas, Rio Grande do Sul, Brasil, de 1982 (22 e 30 anos) e 1993 (22 anos).

	Masculino	Feminino	Diferença absoluta (p.p.)	Razão de prevalência (IC95%) *	Valor de p *
	% (IC95%)	% (IC95%)			
IDF					
Coorte de 1993					
22 anos	19,7 (17,8; 21,6)	16,8 (15,0; 18,5)	2,9	1,17 (1,02; 1,36)	0,024
Coorte de 1982					
22 anos	13,7 (12,1; 15,3)	8,8 (7,6; 10,1)	4,9	1,56 (1,29; 1,87)	< 0,001
30 anos	22,0 (20,0; 23,9)	9,2 (7,8; 10,6)	12,8	2,39 (2,00; 2,82)	< 0,001
NCEP-ATP III					
Coorte de 1993					
22 anos	14,6 (12,8; 16,3)	12,5 (11,0; 14,1)	2,1	1,16 (0,98; 1,37)	0,083
Coorte de 1982					
22 anos	10,7 (9,3; 12,1)	6,5 (5,4; 7,6)	4,2	1,65 (1,33; 2,60)	< 0,001
30 anos	15,0 (13,3; 16,7)	6,5 (5,4; 7,7)	8,5	2,30 (1,87; 2,84)	< 0,001

IC95%: intervalo de 95% de confiança; IDF: Federação Internacional de Diabetes; NCEP-ATP III: *National Cholesterol Education Program Adult Treatment Panel III.*

* Regressão de Poisson com variância robusta.

Foram identificadas desigualdades socioeconômicas na ocorrência de síndrome metabólica em relação a sexo e cor da pele de acordo com o Equiplot. Em todos os acompanhamentos, foi observada maior prevalência de síndrome metabólica entre mulheres de posição socioeconômica mais baixa. Por outro lado, foi verificada maior prevalência entre homens de posição socioeconômica mais alta (Figura 1). Adicionalmente, quando a amostra foi estratificada por cor da pele, foi observada maior desigualdade na prevalência de síndrome metabólica de acordo com a posição socioeconômica entre os indivíduos de cor da pele preta/parda. Tal diferença pôde ser evidenciada pela maior distância entre os pontos de prevalência das posições socioeconômicas mais altas e mais baixas, sobretudo entre os homens pretos/pardos da coorte de 1993 e em homens e mulheres do acompanhamento de 30 anos (coorte de 1982) (Figura 1).

**Figura 1:** Equiplot com prevalência de síndrome metabólica de acordo com posição socioeconômica de acordo com a classificação da Associação Brasileira de Empresas de Pesquisa (ABEP) categorizada em AB, C e DE ^36^, sexo e cor de pele.

IDF: Federação Internacional de Diabetes; NCEP-ATP III: *National Cholesterol Education Program Adult Treatment Panel III.*

As medidas de desigualdade absoluta (SII) e relativa (CIX) confirmaram esse padrão (Tabela 4). Aos 22 anos (coorte de 1982), mulheres de posição socioeconômica mais baixa apresentaram SII de -8,7p.p. e CIX de -20,1%, em contraste com os homens que tiveram SII de 6,5p.p. e CIX de 7,9%. Aos 30 anos (coorte de 1982), esse padrão foi mantido, com mulheres apresentando SII de -6,7p.p. e CIX de -14,8%, e homens, SII de 5,7p.p. e CIX de 5,1% (Tabela 4). Foram identificados padrões semelhantes aos 22 anos na coorte de 1993, maior prevalência de síndrome metabólica foi observada entre mulheres de posição socioeconômica mais baixa (SII: -8,5p.p. e CIX: -12,9%), e entre homens de posição socioeconômica mais alta SII de 9,6p.p. e CIX de 7,3% (critério NCEP-ATP III) (Tabela 4).

**Tabela 4:** Desigualdades absoluta e relativa na prevalência de síndrome metabólica segundo posição socioeconômica *, pelos critérios da *National Cholesterol Education Program Adult Treatment Panel III* (NCEP-ATP III) e da Federação Internacional de Diabetes (IDF, acrônimo em inglês), avaliadas pelo índice de inclinação da desigualdade (SII) e índice de concentração (CIX), nas coortes de nascimento de Pelotas, Rio Grande do Sul, Brasil, de 1982 (22 e 30 anos) e 1993 (22 anos).

	IDF		NCEP-ATP III	
	SII (p.p.) (IC95%)	CIX (IC95%)	SII (p.p.) (IC95%)	CIX (IC95%)
	Coorte de 1993 (22 anos)			
Total	-0,8 (-5,7; 4,0)	-1,9 (-6,0; 2,2)	0,5 (-3,8; 4,7)	-2,0 (-6,8; 2,8)
Brancos	-5,8 (-12,3; 0,7)	0,2 (-0,4; 0,8)	-2,5 (-8,2; 3,3)	0,4 (-0,2; 1,1)
Pretos/Pardos	4,6 (-4,0; 13,2)	0,3 (-0,7; 1,4)	1,5 (-5,9; 8,8)	0,2 (-0,9; 1,2)
Mulheres	-10,3 (-16,7; -3,8) **	-9,4 (-0,3; -3,5) **	-8,5 (-14,2; -2,8) **	-12,9 (-19,6; -6,2) ***
Brancas	-14,3 (-22,9; -5,7) **	-11,5 (-19,0; -4,1) **	-11,5 (-19,2; -3,7) **	-11,8 (-20,2; -3,5) **
Pretas/Pardas	-9,6 (-21,2; 2,1)	-5,7 (-16,1; 4,8)	-11,1 (-21,1; -11,7) **	-13,3 (-25,7; -0,9) **
Homens	8,6 (1,4; 15,9) **	4,2 (-1,4; 9,8)	9,6 (3,2; 15,9) **	7,3 (0,9; 13,8) **
Brancos	2,8 (-7,1; 12,8)	0,0 (-6,8; 6,9)	7,0 (-1,8; 15,7)	4,2 (-3,8; 12,2)
Pretos/Pardos	19,3 (7,1; 31,6) **	13,1 (2,6; 23,5) **	14,4 (4,0; 24,8) **	13,5 (1,3; 25,6) **
	Coorte de 1982 (22 anos)			
Total	-1,8 (-5,9; 2,3)	1,3 (-4,0; 6,6)	-1,1 (-4,8; 2,6)	1,5 (-4,7; 7,8)
Brancos	-2,0 (-6,8; 2,8)	0,6 (-0,2; 1,4)	-1,2 (-5,5; 3,1)	0,6 (-0,1; 1,4)
Pretos/Pardos	-3,4 (-12,2; 5,5)	-0,8 (-2,6; 0,9)	-2,2 (-10,0; 5,5)	-0,8 (-2,5; 1,0)
Mulheres	6,2 ( -0,7; 13,1)	-16,4 (-24,7; -8,2) ***	-8,7 (-13,2; -4,2) ***	-20,1 (-29,7; -10,5) ***
Brancas	7,9 (-0,2; 15,9)	-22,1 (-31,4; -12,7) ***	-9,6 (-14,8; -4,3) ***	-24,2 (-0,35; -13,5) ***
Pretas/Pardas	-2,3 (-15,9; 11,4)	-0,3 (-16,8; 16,2)	-7,7 (-18,1; 2,7)	-7,3 (-28,0; 13,3)
Homens	-9,8 (-14,8; -4,7) ***	7,0 (0,6; 13,5) **	6,5 (0,1; 12,9) **	7,9 (0,6; 15,3) *
Brancos	11,4 (17,4; 5,4) ***	9,3 (1,9; 16,8) **	7,9 (3,1; 15,4) **	9,7 (1,1; 18,3) *
Pretos/Pardos	-4,4 (-15,9; 7,2)	1,6 (-14,0; 17,1)	3,2 (-8,5; 14,8)	6,1 (-10,6; 22,7)
	Coorte de 1982 (30 anos)			
Total	15,5 (-3,2; 6,3)	1,0 (-3,6; 5,5)	0,1 (-3,9; 4,1)	0,6 (-5,0; 6,2)
Brancos	-1,2 (-6,8; 4,3)	0,2 (-0,3; 0,7)	-1,4 (-6,1; 3,3)	0,2 (-0,3; 0,7)
Pretos/Pardos	3,7 (-6,4; 13,8)	-0,2 (-1,4; 1,0)	1,0 (-7,7; 9,7)	-0,3 (-1,5; 9,4)
Mulheres	-5,8 (-11,1; -0,5) **	-10,5 (-19,4; -1,6) **	-6,7 (-11,2; -2,2) *	-14,8 (-25,5; -4,2) **
Brancas	-6,5 (-12,5; -0,4) **	-11,6 (-21,4; -1,8) **	7,4 (-12,6; -2,2) *	-17,3 (-29,0; -5,2) **
Pretas/Pardas	-11,3 (-25,0; 2,4)	-17,6 (-38,8; 3,6)	-9,7 (-20,8; 1,3)	-19,1 (-42,5; 4,4)
Homens	6,7 (-1,0; 14,5)	3,5 (-1,6; 8,6)	5,7 (-1,0; 12,5)	5,1 (-1,2; 11,4)
Brancos	1,6 (-0,8; 11,0)	2,1 (-3,5; 7,8)	3,7 (-4,7; 11,6)	3,9 (-3,1; 10,9)
Pretos/Pardos	1,4 (-0,8; 29,3)	10,6 (-2,4; 23,5)	8,8 (-4,8; 22,3)	14,6 (-1,4; 30,6)

IC95%: intervalo de 95% de confiança.

* Classificação da posição socioeconômica de acordo com Associação Brasileira de Empresas de Pesquisa (ABEP) categorizadas em AB, C e DE ^(36)^;

** Valor de p < 0,05;

*** Valor de p < 0,001.

A análise das diferenças absolutas (SII) e relativas (CIX) por cor da pele revelou maior prevalência de síndrome metabólica entre mulheres de cor da pele branca e posição socioeconômica inferior, sendo essa diferença mais acentuada aos 22 anos (coorte de 1993) pelo critério IDF (SII: -14,3p.p. e CIX: -11,5%). Entre os homens, o padrão variou de acordo com a coorte. Na coorte de 1982, aos 22 anos foi observada maior prevalência de síndrome metabólica entre brancos de posição socioeconômica superior (SII: 11,4p.p. e CIX: 9,3%) (Tabela 4), enquanto na coorte de 1993 (22 anos) foi observada maior prevalência de síndrome metabólica entre homens pretos/pardos também de posição socioeconômica superior (SII: 19,3p.p. e CIX: 13,1%).

## Discussão

A prevalência de síndrome metabólica foi consistentemente mais alta ao se adotar o critério IDF. A síndrome metabólica já afetava 18,2% dos participantes aos 22 anos, ocorrência superior à observada na coorte de 1982 quando aos 22 anos foi de 11,3%, e aos 30 anos 15,5%. Maior prevalência de síndrome metabólica foi apresentada pelos homens em todos os acompanhamentos. A alteração metabólica com maior diferença absoluta (37p.p.) entre os sexos foi a pressão elevada, aos 22 anos (coorte de 1993) (homens 57,8% *vs*. mulheres 20,8%). Ao estratificar por sexo, o critério NCEP-ATP III revelou maior desigualdade por posição socioeconômica na coorte de 1982 aos 22 anos, onde os mais acometidos eram as mulheres de baixa posição socioeconômica e homens de alta (mulheres SII: -8,7p.p. e CIX: -20,1% *vs*. homens SII: 6,5p.p. e CIX: 7,9%). Ao estratificar por sexo e cor da pele, destacou-se que, entre os homens aos 22 anos, a maior prevalência foi apresentada por aqueles de posição socioeconômica superior (pretos/pardos na coorte de 1993 e brancos na coorte de 1982). Já entre as mulheres, o padrão inverteu-se em todos os acompanhamentos avaliados, com a maior prevalência concentrada naquelas de cor da pele branca e posição socioeconômica inferior.

A prevalência da síndrome metabólica aumenta com a idade ^19^ ^,^ ^39^ ^,^ ^40^, o que corrobora com os achados da coorte de 1982. No entanto, a maior prevalência encontrada na coorte de 1993, quando comparada à coorte de 1982 na mesma idade, sugere um possível efeito de coorte, ou seja, decorrente de exposições distintas vivenciadas por grupos nascidos em diferentes períodos - como mudanças alimentares, urbanização, níveis de atividade física e acesso aos serviços de saúde ^41^. Esses achados devem ser interpretados à luz das transições nutricional e epidemiológica ocorridas no Brasil, que tem antecipado o aparecimento das doenças cardiometabólicas ^24^ ^,^ ^41^. Além disso, as diferenças entre as coortes refletem mudanças no sistema de saúde brasileiro. Enquanto os participantes da coorte de 1982 atingiram a vida adulta antes da consolidação do Sistema único de Saúde (SUS), os da coorte de 1993 foram beneficiados pela expansão da atenção primária e de ações preventivas, embora o acesso e a qualidade dos serviços ainda variem segundo posição socioeconômica e contexto social ^24^ ^,^ ^25^ ^,^ ^34^ ^,^ ^35^ ^,^ ^42^ ^,^ ^43^.

A prevalência de síndrome metabólica foi maior entre homens, diferença que pode ser atribuída a mecanismos biológicos e comportamentais ^40^ ^,^ ^44^ ^,^ ^45^. Biologicamente, homens tendem a acumular gordura visceral, enquanto as mulheres acumulam mais gordura subcutânea, influenciados por testosterona e estrogênio, respectivamente ^40^. Adicionalmente, homens e mulheres possuem hábitos de vida distintos. Homens apresentam maior consumo de tabaco ^46^ e alimentação com quantidades excessivas de sal e gordura ^45^. Por sua vez, mulheres apresentam maior consumo de frutas e vegetais, o que pode ter contribuído para essa disparidade ^44^ ^,^ ^45^ ^,^ ^47^. A ingestão excessiva de sal pelos homens pode justificar a diferença dos percentuais de pressão arterial elevada entre os sexos, pois o sal de cozinha, devido seu excesso de sódio, contribui para o aumento da pressão arterial ^48^.

A literatura apresenta resultados heterogêneos sobre a associação entre sexo e síndrome metabólica ^19^ ^,^ ^39^ ^,^ ^49^ ^,^ ^50^. Estudos conduzidos em países de alta renda mostraram que homens de posição socioeconômica mais favorecida apresentam maior prevalência de síndrome metabólica, enquanto, entre aqueles com posição socioeconômica menos favorecidas, a síndrome metabólica é mais frequente em mulheres, sobretudo pretas ou pertencentes a grupos étnicos minoritários ^19^. Nossos achados alinham-se, em parte, com os resultados encontrados nesses países. Observamos maior prevalência de síndrome metabólica entre homens de posição socioeconômica mais alta e em mulheres de posição socioeconômica mais baixa. Padrão que pode ser, pelo menos em parte, reflexo de diferenças comportamentais. Homens de estratos sociais mais altos, em nossa cultura, tendem a ter um estilo de vida mais sedentário, com menor demanda de atividade física principalmente no ambiente de trabalho ^27^ ^,^ ^51^ e maior consumo de gorduras, sal e alimentos ultraprocessados ^52^. 

Como afirmado anteriormente, entre as mulheres, a maior prevalência de síndrome metabólica foi nas de posição socioeconômica mais baixa. Indivíduos de posição socioeconômica inferior tendem a adotar comportamentos menos saudáveis, com maior prevalência de tabagismo, consumo excessivo de álcool, dieta pobre em alimentos saudáveis e menor atividade física de lazer; fatores que favorecem o desenvolvimento de síndrome metabólica ^22^ ^,^ ^53^. Além disto, apresentam acesso desigual a serviços de saúde, com indivíduos de maior renda tendo mais chances de receber cuidados de melhor qualidade, o que também pode impactar na maior prevalência de síndrome metabólica ^23^ ^,^ ^54^. Um resultado inesperado foi a maior prevalência de síndrome metabólica entre as mulheres brancas de baixa posição socioeconômica. Esse resultado pode ser atribuído a fatores de confundimento não mensurados neste estudo descritivo, como por exemplo local de moradia ou acesso a políticas inclusivas, ou ainda ser decorrente da autodeclaração da cor da pele. A mudança na identificação racial observada nos censos mais recentes, onde mais indivíduos se autodeclaram pretos/pardos, sugere que as prevalências anteriores de cor da pele branca podem ter sido superestimadas ^30^. Ainda, embora pretos e pardos de baixa renda enfrentem grandes desafios, é possível que, em alguns contextos, o acesso a redes de apoio social e políticas inclusivas possa atenuar, ainda que parcialmente, os impactos negativos da baixa posição socioeconômica na saúde ^42^. 

Outro resultado surpreendente foi a maior prevalência de síndrome metabólica entre os homens pretos/pardos de posição socioeconômica mais elevada da coorte de 1993, aos 22 anos. Esse achado corrobora com evidências de que indivíduos negros, mesmo após ascensão social, nem sempre experimentam os mesmos ganhos em saúde e qualidade de vida que pessoas brancas na mesma posição socioeconômica ^55^, podendo, em alguns casos, apresentar piora em indicadores de saúde física e mental ^56^.

As desigualdades sociais são determinantes centrais de saúde, operando por mecanismos estruturais e culturais que ampliam a vulnerabilidade a agravos como a síndrome metabólica ^25^. Além dos fatores biológicos e comportamentais, padrões inesperados de síndrome metabólica podem resultar da interação entre determinantes sociais estruturais, que moldam as condições de vida e o acesso a recursos ^24^ ^,^ ^57^; dos processos de corporificação, que traduzem essas desigualdades em alterações biológicas ao longo do tempo ^28^; e das posições interseccionais que modulam de forma desigual as exposições e vulnerabilidades na população ^58^. Os determinantes sociais analisados neste estudo (posição socioeconômica, cor da pele e sexo) apresentam padrões amplamente descritos, com maior vulnerabilidade entre as pessoas de menor renda e escolaridade devido maior exposição a fatores de risco ambientais, alimentares e ocupacionais além de menor acesso a serviços ^24^. Tanto a cor da pele quanto o sexo definem experiências de descriminação, hierarquia de oportunidades, estrutura papeis sociais e trajetórias laborais, impondo diferentes cargas de trabalho e renda ^58^. Essas desigualdades, sob a perspectiva ecossocial ^28^, tornam-se corporificados ao longo da vida, ou seja, as desigualdades sociais e ambientais são literalmente incorporadas pelo corpo e expressas em diferenças biológicas e metabólicas, o que poderia justificar a maior prevalência de síndrome metabólica em homens pretos/pardos de posição socioeconômica mais elevada da coorte de 1993, aos 22 anos. Essas intersecções entre cor da pele, posição socioeconômica e sexo produzem exposições diferenciadas e vulnerabilidades específicas manifestando-se biologicamente em perfis metabólicos distintos entre os grupos sociais como as que foram observadas nesse estudo.

Dessa forma, a formulação de políticas públicas eficazes requer o enfrentamento dos determinantes sociais que sustentam as desigualdades em saúde, superando a ênfase nas “escolhas individuais”. Estratégias voltadas à síndrome metabólica devem considerar a redução das iniquidades socioeconômicas, cor da pele e de sexo e a criação de condições sociais que possibilitem escolhas saudáveis e equitativas.

Este estudo apresenta algumas limitações. A primeira refere-se às perdas de acompanhamento, comum em estudos de coorte. Os indivíduos acompanhados representaram cerca de 70% da coorte inicial, percentual considerado satisfatório para estudos de base populacional. Houve diferença por sexo, sendo maior o acompanhamento das mulheres. Como as análises foram estratificadas por cor da pele e sexo, esse viés tende a ser minimizado. É necessário ainda enfatizar que as coletas analisadas ocorreram nos anos de 2004, 2012 e 2015 e, portanto, as prevalências apresentadas podem não refletir integralmente os níveis atuais de síndrome metabólica na população.

A segunda limitação é sobre a aferição da pressão aos 22 anos na coorte de 1982, que foi realizada com esfigmomanômetro digital de pulso, método diferente dos outros dois acompanhamentos. Embora as medidas por esse tipo de aparelho sejam comparáveis às de um aparelho de mercúrio, elas tendem a ser ligeiramente superiores ^59^. Dessa forma, é possível que a ocorrência de pressão elevada possa ter sido superestimada nesse acompanhamento. A terceira limitação refere-se à coleta dos exames laboratoriais séricos e da glicemia capilar pois foi realizada ao acaso o que pode ter superestimado a glicemia e em menor proporção os triglicerídeos e HDL, visto que o jejum não é mandatório para avaliação do perfil lipídico ^60^.

Por fim, aos 22 anos (coorte de 1982) a glicemia capilar foi aferida com o aparelho Accu-Chek Advantage (Roche, https://www.roche.com.br/) que, apesar de possuir boa sensibilidade (93,8%) e especificidade (83,6%) ^61^, tende a superestimar a glicemia ^62^. Esse fator pode ter aumentado artificialmente a prevalência de síndrome metabólica nesse grupo. Como esse foi o acompanhamento com a menor prevalência geral, a diferença real entre as duas coortes aos 22 anos provavelmente é ainda maior do que a observada.

No acompanhamento de 22 anos (coorte de 1993), a definição de diabetes, HAS e dislipidemia incluiu o diagnóstico médico autorreferido e o uso de medicamentos, o que não ocorreu nos demais acompanhamentos. A análise de concordância entre as duas definições (com e sem autorrelato) foi perfeita (kappa = 1,0) para as três condições, indicando que a inclusão do autorrelato não alterou a prevalência das doenças nem introduziu viés nas análises. 

A padronização da coleta da grande maioria dos dados e rigor metodológico no tratamento das informações são pontos fortes do estudo. Outros pontos fortes foram a abordagem interseccional das desigualdades e a utilização de diferentes indicadores para avaliação das desigualdades, tanto de forma relativa quanto absoluta, que evidenciaram um padrão consistente de desigualdades em todos os acompanhamentos analisados. O uso de duas coortes para análise também contribui para a consistência e robustez dos resultados.

Neste estudo foram identificados padrões distintos de síndrome metabólica conforme posição socioeconômica, sexo e cor da pele. Observamos maior prevalência de síndrome metabólica em homens de estratos sociais superiores, principalmente de cor da pele preta/parda, e em mulheres de estratos sociais inferiores, sobretudo de cor de pele branca. Sugerimos a realização de novos estudos que aprofundem a compreensão dos processos e mecanismos envolvidos nas desigualdades da ocorrência de síndrome metabólica na população brasileira.

## Data Availability

As fontes de informação utilizadas no estudo estão indicadas no corpo do artigo.
